# Natural Genetic Variation of Integrin Alpha L (*Itgal*) Modulates Ischemic Brain Injury in Stroke

**DOI:** 10.1371/journal.pgen.1003807

**Published:** 2013-10-10

**Authors:** Sehoon Keum, Han Kyu Lee, Pei-Lun Chu, Matthew J. Kan, Min-Nung Huang, Carol J. Gallione, Michael D. Gunn, Donald C. Lo, Douglas A. Marchuk

**Affiliations:** 1 Department of Molecular Genetics and Microbiology, Duke University Medical Center, Durham, North Carolina, United States of America; 2 Department of Immunology, Duke University Medical Center, Durham, North Carolina, United States of America; 3 Department of Medicine, Duke University Medical Center, Durham, North Carolina, United States of America; 4 Center for Drug Discovery and Department of Neurobiology, Duke University Medical Center, Durham, North Carolina, United States of America; National Cancer Institute, United States of America

## Abstract

During ischemic stroke, occlusion of the cerebrovasculature causes neuronal cell death (infarction), but naturally occurring genetic factors modulating infarction have been difficult to identify in human populations. In a surgically induced mouse model of ischemic stroke, we have previously mapped *Civq1* to distal chromosome 7 as a quantitative trait locus determining infarct volume. In this study, genome-wide association mapping using 32 inbred mouse strains and an additional linkage scan for infarct volume confirmed that the size of the infarct is determined by ancestral alleles of the causative gene(s). The genetically isolated *Civq1* locus in reciprocal recombinant congenic mice refined the critical interval and demonstrated that infarct size is determined by both vascular (collateral vessel anatomy) and non-vascular (neuroprotection) effects. Through the use of interval-specific SNP haplotype analysis, we further refined the *Civq1* locus and identified integrin alpha L (*Itgal*) as one of the causative genes for *Civq1*. *Itgal* is the only gene that exhibits both strain-specific amino acid substitutions and expression differences. Coding SNPs, a 5-bp insertion in exon 30b, and increased mRNA and protein expression of a splice variant of the gene (*Itgal*-003, ENSMUST00000120857), all segregate with infarct volume. Mice lacking *Itgal* show increased neuronal cell death in both *ex vivo* brain slice and *in vivo* focal cerebral ischemia. Our data demonstrate that sequence variation in *Itgal* modulates ischemic brain injury, and that infarct volume is determined by both vascular and non-vascular mechanisms.

## Author Summary

Stroke is the second leading cause of death and the most common cause of acquired adult disability worldwide. Ischemic stroke is caused by an interruption of blood flow in the cerebral arteries and results in neuronal damage (infarct) to the area of perfusion in the brain. Although significant progress has been made in the identification of genetic risk factors for stroke susceptibility, identification of genetic factors determining the severity of tissue damage has proven more challenging. By contrast, infarct volume varies widely among laboratory inbred mouse strains. Using a well-established mouse model of stroke and complex genetic analysis, we have exploited these differences and identified *Itgal* as one of the candidate genes. We further show that allelic variation in *Itgal* segregates with infarct volume among inbred mouse strains and deficiency of the gene increases ischemic neuronal cell death in stroke.

## Introduction

Stroke is the second leading cause of death and the most common cause of acquired adult disability worldwide [1], [2]. Ischemic stroke is caused by disrupted blood flow within the territory of an occluded blood vessel that results in death of brain cells (infarct). The severity of cerebral infarction primarily depends on the re-perfusion and response of the blood vessels, but neuronal cell death is also determined by intrinsic molecular cascades including excitotoxicity, oxidative stress, apoptosis, and inflammation [3]. More recently, emerging data suggest that the dynamic interaction between vascular cells, glia, neurons and associated tissue matrix proteins – the neurovascular unit – plays a crucial role in the pathogenesis of ischemic brain injury [4].

Although genome-wide association studies have made some progress in the identification of stroke susceptibility genes [5], the genetic determinants for stroke outcome have yet to be fully explained. Because variation in the anatomic location of the occluded artery, the extent and duration of occlusion, time until treatment, and other contributing factors cannot be controlled in patients, very few genetic factors have been identified that contribute to the severity of brain damage in human ischemic stroke. By contrast, infarct volume in murine models of focal cerebral ischemia (stroke) has been shown to vary widely among inbred strains, suggesting strong genetic control [6]–[8]. In a mouse model of ischemic stroke, we have previously demonstrated that infarct volume varies up to 30-fold in 16 common inbred mouse strains. Using a forward genetic mapping analysis in an F2 intercross between C57BL/6J (B6 hereafter) and BALB/cByJ (BALB/c) strains, we have identified three distinct quantitative trait loci (QTLs) that modulate the volume of cerebral infarction. In particular, a single locus mapping to distal chromosome 7, *Civq1* (cerebral infarct volume QTL1), accounts for the major portion of variation (56%) in infarct volume [8]. In the present study, through the use of multiple QTL mapping analyses, generation of sub-congenic mouse lines, genome-wide association across inbred strains, and ancestral SNP haplotype analyses, we have identified that genetic variation in integrin alpha L (*Itgal*) modulates ischemic brain injury in mice.

## Results

### Characterization of cerebral infarct volumes in 32 different inbred mouse strains

**Figure 1:**
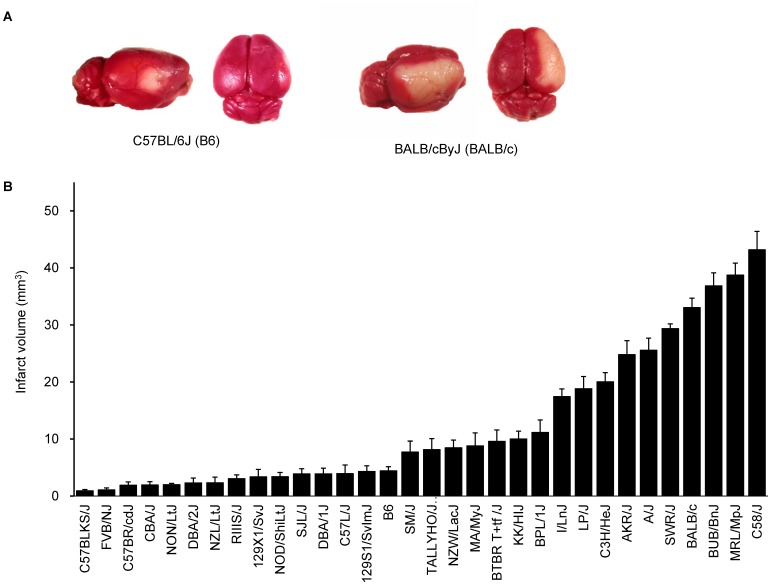
Wide phenotypic variation in infarct volume in 32 inbred mouse strains. A. Representative topical TTC-stained brains from B6 and BALB/c mice showing the cortical infarct 24 hr after distal MCAO. B. Distribution of infarct volume across 32 inbred mouse strains. Values represent mean ± SEM for at least 5 animals per strain.

Using 16 common inbred mouse strains, we have previously demonstrated that infarct volume after permanent distal middle cerebral artery occlusion (MCAO) is under strong genetic control, and had mapped *Civq1* on chromosome 7 as a major genetic determinant of infarct volume [8]. To further explore the naturally occurring genetic variation in infarct volume, and provide additional statistical power and map resolution, we determined infarct volumes on additional 16 inbred strains, representing the priority strains for the Mouse Phenome Project (http://phenome.jax.org). We excluded wild-derived strains to avoid spurious false positive association [9]–[11]. The ischemic damage was localized exclusively in the frontal and parietal cortex and infarct volumes were highly reproducible among individual animals of the same inbred strain. Similar to our previous report [8], we observed large variability in infarct volumes among strains (Figure 1A,B). Strains I/LnJ, LP/J, C3H/HeJ, AKR/J, A/J, BALB/c, SWR/J (SWR), BUB/BnJ, MRL/MpJ, and C58/J exhibited marked sensitivity to ischemic injury and developed large infarct volumes (>17.0 mm^3^). By contrast, strains C57BLKS/J, FVB/NJ (FVB), C57BR/cdJ, CBA/J, NON/LtJ, DBA/2J, NZL/LtJ, RIIIS/J, 129X1/SvJ, NOD/ShiLtJ, SJL/J, DBA/1J, C57L/J, B6, and 129S1/SvImJ were relatively resistant to cerebral ischemia, showing infarct volume smaller than 5 mm^3^ (Figure 1B). Mean infarct volume ranged from 0.9 to 43.0 mm^3^ between the strain pairs at the phenotypic extremes (C57BLKS/J vs. C58/J), representing a 47-fold difference in the trait.

### Genome-wide association study remapped and reduced *Civq1*

**Figure 2:**
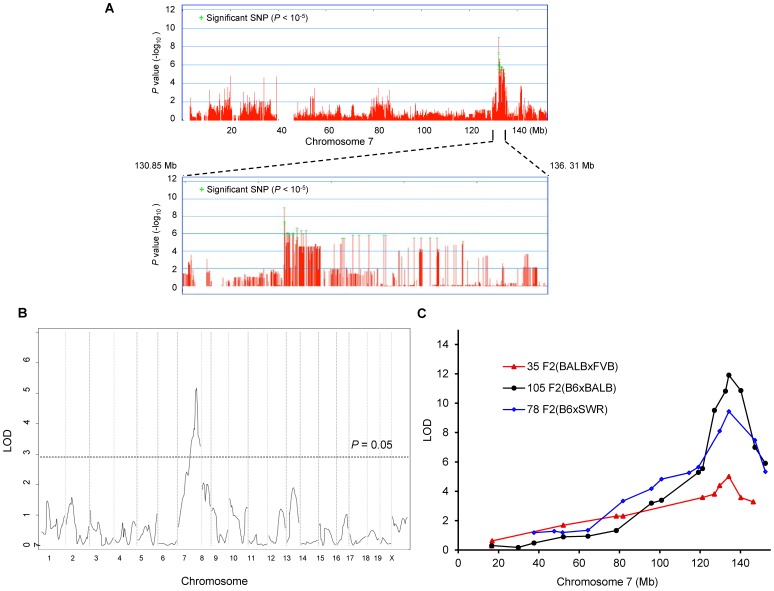
*Civq1* on chromosome 7 re-identified in intercrosses between multiple mouse strains. A. Genome-wide EMMA analysis across chromosome 7 for cerebral infarct volume in 32 inbred mouse strains. The plot is drawn for −log(P) against SNP positions on the chromosome. Green crosses represent SNPs over the significance threshold of P = 10^−5^. The genomic region (132.35–134.81 Mb) with significant association in chromosome 7 maps within *Civq1* detected by multiple linkage studies. B. The graph presents the results of a genome-wide linkage scan for infarct volume in 35 (FVB×BALB) F2 progeny. Chromosomes 1 through X are represented numerically on the x-axis and the y-axis represents the LOD score. The significant (*P<0.05*) level of linkage is determined by 1,000 permutation tests. Only a single genomic region on chromosome 7 displays significant linkage to infarct volume with a LOD score of 5.2. C. *Civq1* is mapped to distal chromosome 7 by linkage analyses in multiple F2 (B6×BALB/c, B6×SWR, and FVB×BALB/c) populations. The same SNP marker (rs13479513) is located at the peaks of all linkage analyses.

From these combined data on 32 total strains (255 total mice), we preformed genome-wide associations for this trait. To correct for population structure and genetic relatedness among inbred strains of mice, we employed the Efficient Mixed Model Association (EMMA) [12]. The association scan was carried out using the 4 million high-density SNP panel using the EMMA server (http://mouse.cs.ucla.edu/emmaserver). We identified a significant region of association (Chr7: 132.35–134.81 Mb) that co-localized within the 95% confidence interval of *Civq1* that we previously mapped in linkage analyses (Figure 2A) [8]. A total of 69 SNPs across the ∼2.5 Mb region reached the statistically significant threshold (*P*<10^−5^) for cerebral infarct volume and the most significant association without any missing alleles was at a SNP (rs32965660, *P* = 1.25×10^−6^) at 132,390,712 bp on the chromosome.

Our EMMA associated region for *Civq1* on chromosome 7 covers approximately 2.5 Mb (132.35–134.81 Mb). A previous report calculated a median distance of 3 Mb between the actual causal variant and the closest marker in EMMA analysis [11], so we expanded our candidate interval for an additional 1.5 Mb region flanking either side of the associated SNPs. There are 124 known genes in the expanded associated region (130.85–136.31 Mb) on chromosome 7 (Figure 2A). Because of lack of precision in genome-wide association studies due to incomplete understanding of linkage disequilibrium in the mouse genome, statistical power is highly dependent on the number and genetic relatedness of the inbred mouse strains used [13], [14]. A previous study suggests that for a trait with a genetic effect contributing in the range of 30% to the total variance (*Civq1* = 56%), 30 strains or more are required for acceptable power [15]. This suggests that our analysis is sufficiently powered, and that the causative gene for the *Civq1* locus is located within the expanded 5.5 Mb region, and most likely, in the 2.5 Mb region, reduced by our EMMA analysis using 32 inbred strains.

### *Civq1* is remapped in an intercross between FVB and BALB/c strains

Since we previously identified *Civq1* in two different genetic crosses (B6×BALB/c and B6×SWR), as well as in the Chromosome Substitution Strain 7 (CSS7) where A/J chromosome 7 was introgressed into the B6 background, and each cross includes B6 as one of the parental strains [8], it is possible that the sequence variant underlying *Civq1* is unique to B6, occurring in this strain only after it was separated from its last common ancestor with the other strains [16]–[19]. To determine whether allelic variation at *Civq1* is unique to the B6 strain or instead due to a sequence variant mapping within an ancestral murine haplotype block [20], we performed an additional intercross between the large infarct strain, BALB/c, and a *different* small infarct strain FVB; two strains which exhibit a 10-fold difference in infarct volume (Figure 1B). By substituting FVB for B6 as the “small infarct” strain in this new cross, we could effectively determine whether FVB and B6 share the “protective” allele at *Civq1*. Because our goal was to determine whether we would remap *Civq1* in this new cross, and to date, *Civq1* had shown effect sizes in excess of 50% in other crosses, we surmised that if *Civq1* was responsible for the difference between these two parental strains, the locus could be identified with a minimum number of F2 animals. Even with only 35 F2 (FVB×BALB/c) mice, we identified a statistically significant locus (LOD = 5.2) that mapped to the identical position (peak LOD at rs13479513) on chromosome 7 as that of *Civq1* (Figure 2B). Interestingly, the locus identified in this F2 (FVB× BALB/c) cross exhibits a large effect size (∼85%), even stronger than observed in our original two crosses (56–57%). In this cross, *Civq1* accounts for nearly all of the phenotypic difference in infarct volume observed between FVB and BALB/c strains (Figure S1), and this may explain the highly significant LOD score obtained with only 35 F2 progeny. These combined data further validate the importance of *Civq1* in the determination of infarct volume across common inbred mouse strains (Figure 2C). More importantly, these data strongly suggest that the sequence variant underlying *Civq1* is located within an ancestral haplotype block that has been inherited across multiple inbred mouse strains, rather than being unique to strain B6. This further supports the use of ancestral haplotype association mapping approaches to fine map the *Civq* locus, towards the identification of the causative gene variant(s).

### Congenic mouse lines retaining the infarct volume phenotype reduce the critical QTL interval and display vascular and non-vascular effects on stroke outcome

**Figure 3:**
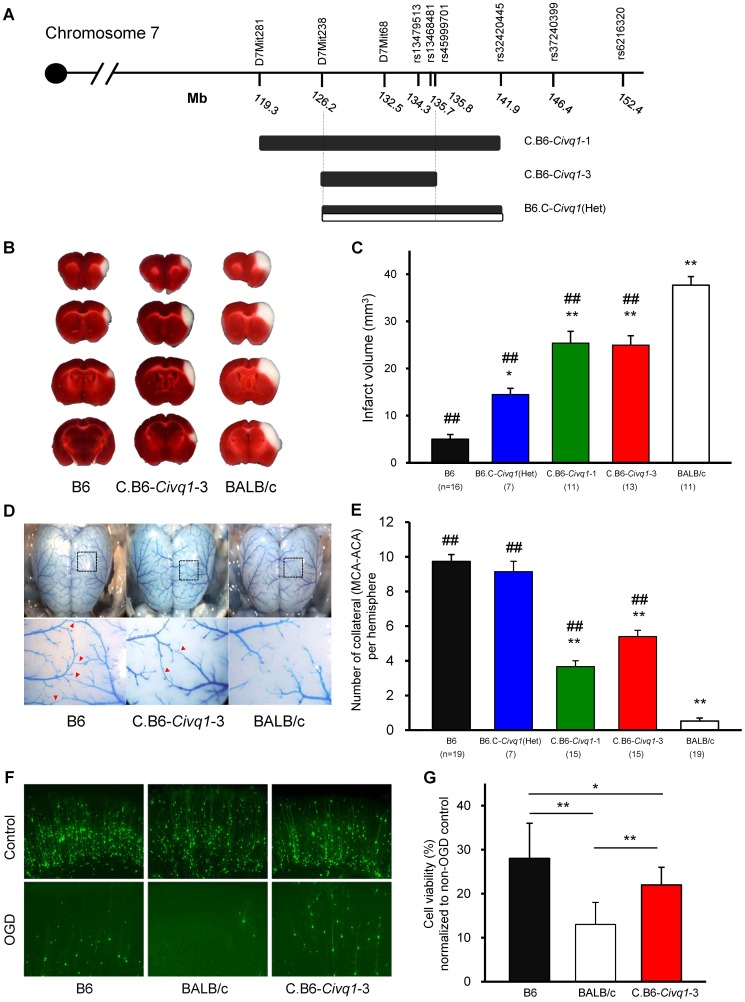
The critical interval of chromosome 7 retains both infarct volume and collateral artery number phenotypes in congenic animals. A. Schematic map of two *Civq1* congenic lines carrying segments of B6 chromosome 7 introgressed into the BALB/c background, and heterozygous BALB/c alleles on B6 background. The latter could only be maintained as a heterozygote with B6. B. The posterior faces of representative 1-mm coronal sections from the B6, C.B6-*Civq1*-3, and BALB/c mice are shown. The size of the infarct is much smaller in C.B6-*Civq1*-3 than control BALB/c mouse. C. Congenic mice (C.B6-*Civq1*-1 and -3) exhibit a reduction in infarct volume by ∼30% compared to BALB/c mice. Infarct volume in congenic mice carrying heterozygous BALB/c alleles on B6 background (B6.C-*Civq1*(HET)) is 2.5-fold larger than that of control B6 mice. ## *P<0.001* vs. BALB/c; *P<0.05, ** *P<0.001* vs. B6. Values represent mean ± SEM. D. Superficial angioarchitecture of the brain in 4-week old B6, C.B6-*Civq1*-3, and BALB/c mice. Magnified images of the box in the upper panels are shown in the lower panels. The red arrowheads indicate the vessels of pial anastomoses between the MCA and ACA. E. C.B6-*Civq1* congenic lines show an increase in the number of collaterals connecting the distal MCA and ACA compared control BALB/c strains. Heterozygous congenic mice show no difference compared to control B6 mice. ## *P<0.001* vs. BALB/c; ** *P<0.001* vs. B6. F. Cortical brain slices from B6, BALB/c, and C.B6-*Civq1*-3 mice biolistically transfected with an YFP expression plasmid under normal conditions and 24 hr after 5.5 min of OGD. G. C.B6-*Civq1*-3 mice exhibit ∼50% increased neuronal viability compared to control BABL/c mice. Total numbers of healthy and YFP-positive neurons in the cortical region of the brain slices were counted at 24 hr after transient OGD. Cell viability was normalized from their non-OGD controls. Values represent mean±SEM from at least 5 animals per group. * *P<0.05* and ** *P<0.001*.

To validate the phenotypic effects of the isolated *Civq1* locus, and to narrow down the critical region of the QTL, we created recombinant congenic mouse lines (C.B6-*Civq1*) carrying different segments of the *Civq1* region from B6 introgressed into the BALB/c background. Congenic Line 1 contains approximately 22.6 Mb of the B6 region of *Civq1*, and Line 3 is a fully nested sub-congenic line containing a smaller region completely contained within the larger congenic region (C.B6-*Civq1*-1: 119.3–141.9 Mb and C.B6-*Civq1*-3: 126.2–135.8 Mb). We also attempted to generate a reciprocal congenic line (B6.C-*Civq1*) containing the *Civq1* region from BALB/c on the B6 background (Figure 3A), but for unknown reasons, the reciprocal congenic line was embryonic lethal when crossed to homozygosity (0 homozygotes out of 52 progeny from a heterozygous congenic cross). Nonetheless, since both *Civq1* exhibit heterozygous effects in the mapping crosses, we retained the heterozygous congenic line (B6.C-*Civq1*(Het)) for analysis (retaining approximately 16 Mb of BALB/c genomic from D7Mit238 to rs32420445).

We first analyzed infarct volume of the congenic lines. Both C.B6-*Civq1*-1 and -3 lines showed significantly reduced (∼30%) volume of cerebral infarction compared to control BALB/c mice (Figure 3B,C). There was no significant difference in infarct volume between the two lines carrying the larger or fully nested, smaller, segment of B6 *Civq1* region, providing evidence that the 9.6 Mb region (126.2–135.8 Mb) located between the markers D7Mit238 and rs45999701 defines the critical interval for *Civq1* (Figure 3A).

In a B6×BALB/c intercross, a locus modulating the number of pial collateral arteries (Collateral artery number QTL, *Canq1*) [21] was mapped that appears to coincide with our infarct volume locus, *Civq1*. This suggested that allelic variation in the same gene(s) might modulate the phenotypes of both infarct volume and collateral vessel formation in the brain. We next determined the collateral artery phenotype of these same congenic lines, measuring pial collateral arteries connecting between MCA and ACA (Figure 3D). Consistent with previous reports [21], BALB/c mice have on average less than one collateral artery per cerebral hemisphere, compared to an average of 10 in B6 mice. Both Lines 1 and 3 of the (C.B6-*Civq1*) congenic lines showed an approximately 50% increase in the number of collateral arteries when compared to control background BALB/c mice, and similar to the infarct volume data, there was no difference between C.B6-*Civq1*-1 and -3 lines (Figure 3E). Surprisingly, although the heterozygous reciprocal congenic mice (B6.C-*Civq1*(Het)) showed no difference in pial collateral number when compared with B6 controls (Figure 3E), the infarct volume of the heterozygous congenic mice was significantly increased when compared to B6 mice (Figure 3C). Infarct volume of the congenic mice (B6.C-*Civq1*(Het)) was ∼3-fold larger than that of B6 mice (14.7 mm^3^ vs. 4.5 mm^3^).

To examine whether the *Civq1* locus confers a collateral-independent, tissue-intrinsic effect on cerebral infarction, we performed the widely used brain slice-based assay where transient oxygen-glucose deprivation (OGD) is used to induce neuronal cell death. Neuronal degeneration was measured via biolistic transfection of the vital fluorescent reporter, YFP, which creates a dispersed ‘sentinel’ population of cortical pyramidal neurons that can be used to quantify neuronal vitality and numbers [22], [23]. We first examined neuronal cell death for parental B6 and BALB/c strains. Subjecting YFP-transfected coronal brain slices to transient OGD resulted in the degeneration and clearance of a large proportion of cortical pyramidal neurons by 24 hr post OGD treatment. Intriguingly, similar to the sensitivity to focal cerebral ischemia, cell viability in YFP-transfected brain slices in B6 mice was significantly higher than that in BALB/c mice (Figure 3F,G). Based on this finding, we next determined the phenotype of the C.B6-*Civq1*-3 congenic mice. The congenic mice displayed a significantly increased number of YFP-positive live cortical neurons in OGD-treated brain slices when compared with control BALB/c mice (40%; Figure 3F,G). There was no difference in YFP transfection efficiency and viability in non OGD-treated brain slices between these mouse strains (Figure S2). In support of differential resistance to OGD in brain tissues between these strains, western blot analysis showed that the level of cleaved Caspase-3 was significantly reduced in lysates of brain slices from C.B6-*Civq1*-3 mice compared to control BALB/c mice after OGD treatment (Figure 3S). Taken together, these results show that the B6 allele(s) of at least one of the causal gene(s) underlying *Civq1* provides non-vascular, tissue-intrinsic resistance to ischemic brain injury. More importantly, although the sum of genetic evidence to date suggested that *Civq1*, regulating infarct volume, and *Canq1*, controlling collateral artery density, are mapped to the identical genomic region, the data from the congenic strain (B6.C-*Civq1*(Het)) and these *ex vivo*, OGD experiments using brain slice explants that lack functioning vasculature suggest that the *Civq1* locus may be more complex than *Canq1*, containing at least one gene variant that modulates ischemic brain injury independent of collateral artery density.

### Interval-specific ancestral SNP haplotype analysis and fine-mapping of *Civq1* toward candidate gene identification

**Figure 4:**
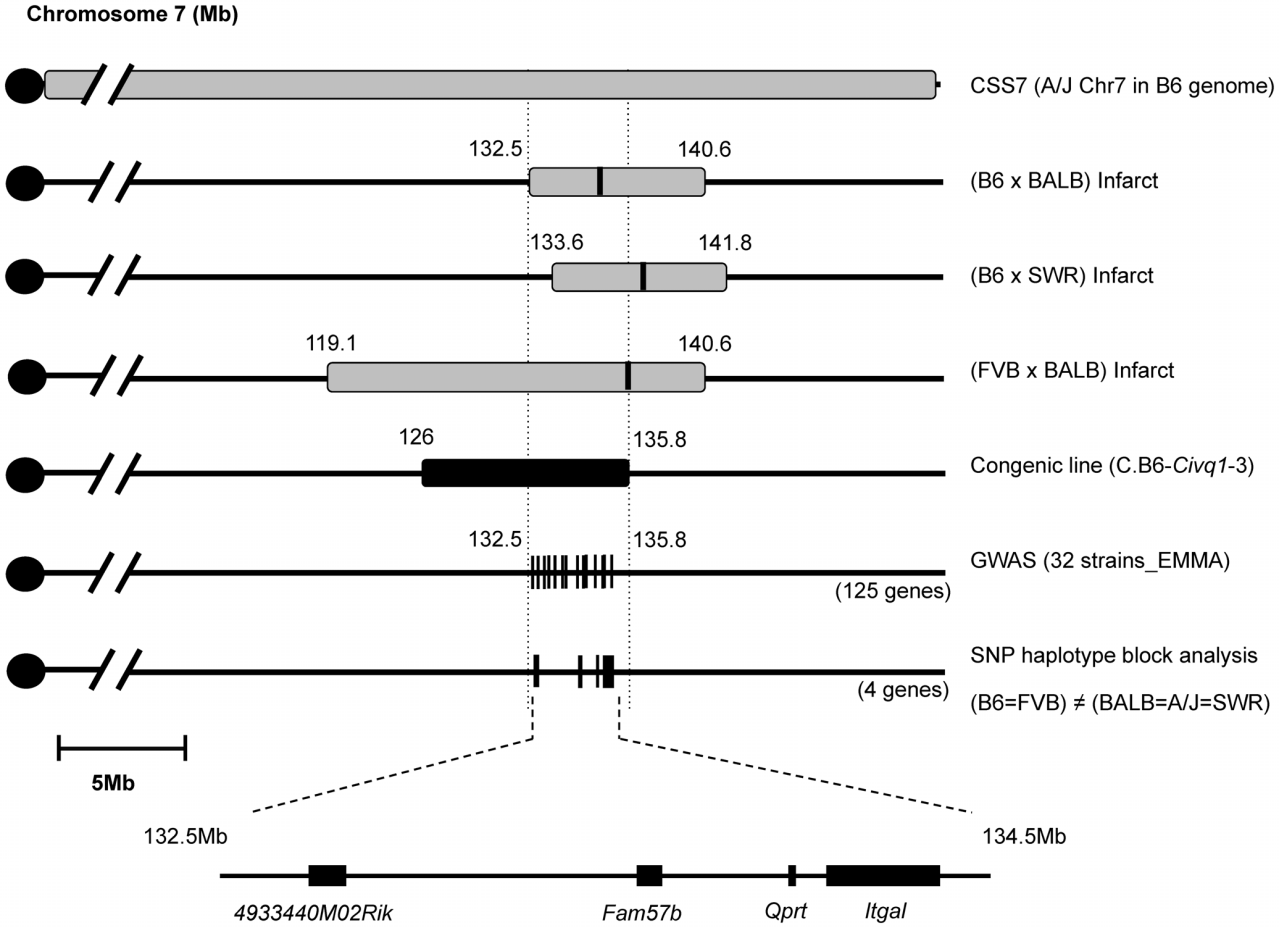
Fine mapping of *Civq1* harboring a causative candidate gene for infarct volume. The map shows chromosome 7 with the previously mapped QTLs and their 95% confidence intervals, CSS7, the critical congenic interval, the genome-wide association peak region, and the interval-specific SNP haplotype block analysis among the 5 mapping strains. The 4 candidate genes are clearly located with SNP haplotype blocks shared between the small infarct strains (B6 and FVB) but differing from the large infarct strains (BALB/c, SWR/J, and A/J).

The congenic line (C.B6-*Civq1*-3) reduces the critical QTL interval to a 9.6 Mb interval between D7Mit238 at 126.2 Mb and rs45999701 at 135.8 Mb, but this region still harbors over 200 genes. Although genome-wide association analysis can be employed to significantly reduce a QTL interval for candidate gene identification [24], the phenotype-associated EMMA interval in this region of chromosome 7 encompasses approximately 2.5 Mb, consisting of a genomic region of unusually high gene density, harboring more than 100 potential candidate genes. To further dissect the interval, we compared ancestral SNP haplotype patterns across the inbred mouse lineages, specifically focusing on those strains for which we had generated independent genetic mapping information [25]–[27]. Interval-specific SNP haplotype block analysis can reduce confidence intervals by identifying high-priority regions within a QTL interval that are likely to harbor the causal polymorphism [27], [28]. Because *Civq1* was identified in three different genetic crosses (B6×BALB/c, B6×SWR, and FVB×BALB/c) and mapped more broadly to chromosome 7 using the CSS (B6×A/J) series, allelic variation at *Civq1* is most likely harbored by a gene that maps within an ancestral haplotype block that is shared between BALB/c, A/J, and SWR (large infarct volumes), but that is different from B6 and FVB strains (small infarct volumes). As illustrated in Figure 4, defining a haplotype block to be three or more adjacent consecutive shared SNP alleles [25], we identified all SNP haplotype blocks throughout the 3.3 Mb critical region of *Civq1* (132.5–135.8 Mb), a region consistent with each of the 95% confidence intervals of the 4 independent linkage peaks for *Civq1*. Only 4 genes (*4933440M02Rik*, *Fam57b* (*1500016O10Rik*), *Qprt*, and *Itgal*) fall within haplotype blocks matching the phenotype pattern of the mapping strains (Figure 4).

### *Itgal* harbors non-synonymous coding SNPs and is differentially expressed between mouse strains

To identify the causal gene for *Civq1*, we first sought the presence of non-synonymous coding SNPs in these genes. Re-sequencing of the 4 candidate genes identified non-synonymous amino acid substitutions in *Qprt* and *Itgal*. *Qprt* encoding quinolinate phosphoribosyltransferase harbors two coding SNPs (E205K and D253N) that segregate with the infarct volume phenotype (Figure S4). These changes occur at residues that are not well conserved between mammalian species and that are predicted to be functionally benign by *in silico* amino acid substitution analysis in the three different databases, PolyPhen (http://genetics.bwh.harvard.edu/pph/), PMut (http://mmb2.pcb.ub.es:8080/PMut/), and Panther (http://www.pantherdb.org/) [29]. *Itgal* (CD11a) encodes an α subunit of β_2_-integrin Lymphocyte Function associated Antigen-1 (LFA-1) that mediates adhesion and migration of leukocytes. The gene harbors two coding SNPs that create W972R and P978L polymorphisms located in the calf-2 extracellular domain of the protein. Strains B6 and FVB that exhibit small infarcts, encode the W972 and P978 isoform, whereas BALB/c, A/J, and SWR strains that exhibit large infarcts, encode the R972 and L978 isoform. Interestingly, despite a lack of conservation at these residues across other species, the W972R change is predicted to be deleterious to the protein in the three *in silico* databases. No coding changes were identified in the two uncharacterized genes, *4933440M02Rik* and *Fam57b*.

**Figure 5:**
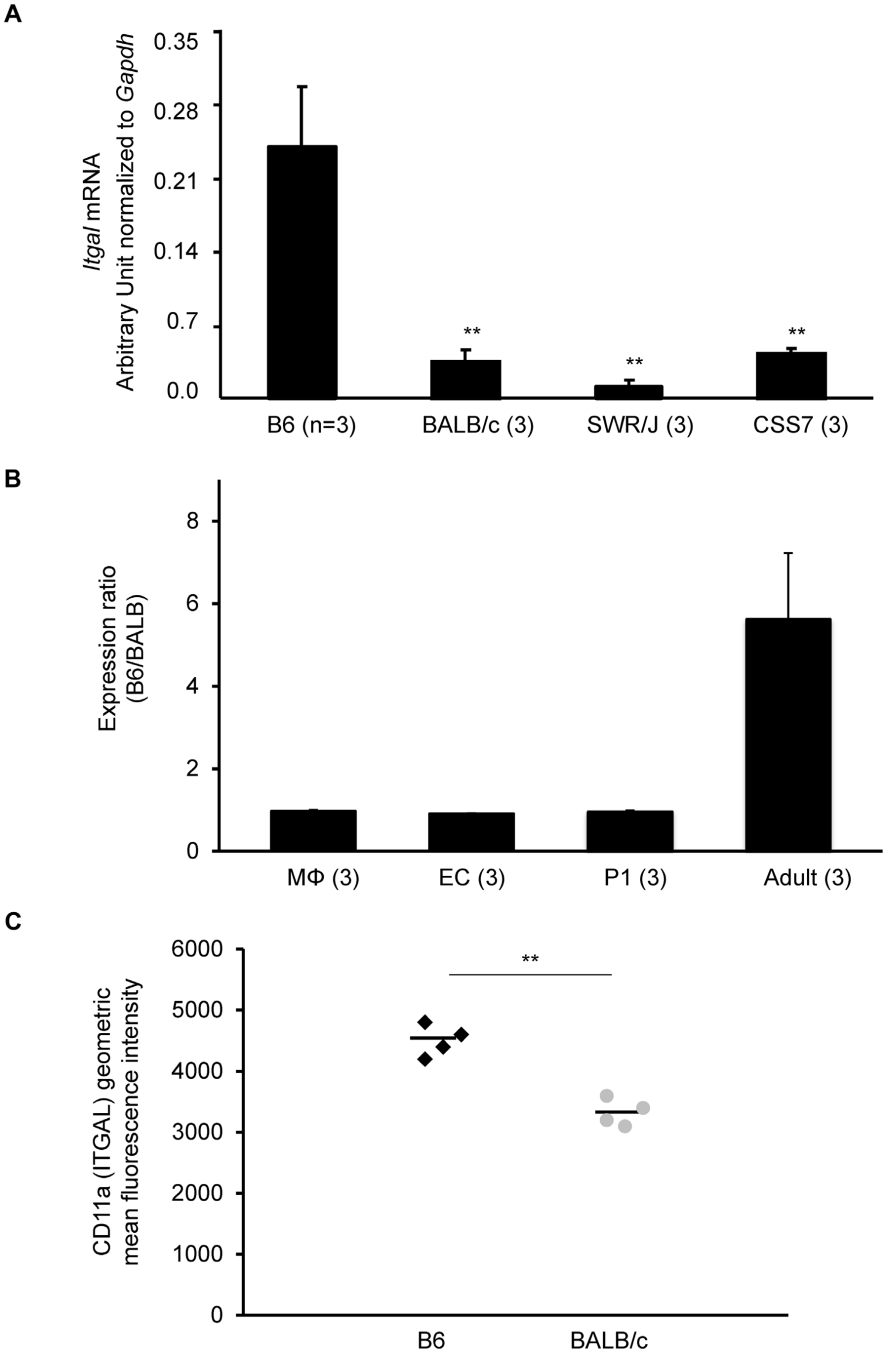
The strain-specific difference in *Itgal* message level is determined by *cis*-acting elements at the *Civq1* locus. A. mRNA levels of the canonical isoform of *Itgal (Itgal-002)* in adult cortex from B6, BALB/c, SWR, and CSS7 strains as determined by qRT-PCR. Each bar represents mean ± SEM. ** *P<0.001* vs. B6. B. The allele-specific expression ratio of *Itgal* transcript (*Itgal*-002) levels in F1 (B6×BALB) mice. A coding SNP (rs107634043) in exon 30 was used to monitor the B6-allele and BALB/c-allele transcripts of *Itgal* in macrophages (MΦ), endothelial cells (EC), P1 and the adult cerebral cortices. The level of the B6-specific *Itgal* transcript was approximately 6-fold higher than BALB/c-transcript in adult cortex. Each bar represents the allele-specific expression ratio (B6/BALB) averaged for three F1 animals. C. Surface expression of CD11a (ITGAL) on CD11a+ brain immune cells. CD45+ CD11a+ brain cells from BALB/c mice express significantly less surface CD11a as assessed by flow cytometric staining than B6 CD45+ CD11a+ brain cells (** *P<0.001*).

Next, to identify genes that show different levels of mRNA between the mapping strains, a surrogate measure of the effects of “regulatory” sequence variants (broadly defined), we performed quantitative real time PCR (qRT-PCR) for the 4 candidate genes. In adult brain cortex, only a single gene, *Itgal*, shows a strain-specific expression difference; an 8-fold higher transcript level in B6 cortex than seen in cortex from the large infarct strains (BALB/c, CSS7, and SWR) (Figure 5A). Since the causative variant for the infarct phenotype would need to reside within the mapped *Civq1* interval, we performed allele-specific gene expression analysis to determine whether the observed expression difference is due to *cis*-acting variation [30]. Similar to the qRT-PCR results, allele-specific gene expression analysis confirmed that the level of B6 *Itgal* transcript is approximately 6-fold higher than BALB/c transcript in the adult cortex in F1 (B6×BALB/c) animals (Figure 5B), providing further evidence that regulatory genetic variation in *cis* causes the difference in *Itgal* mRNA abundance in the brain. These differences at the mRNA level were also seen at the protein level, as detected by flow cytometry of CD45-positive cells isolated from adult brain. We found that the level of ITGAL protein is significantly higher in B6 than in BALB/c mice (Figure 5C). Because the *Civq1* locus contains at least one gene that modulates infarct volume via effects on collateral artery formation [21], we also examined the mRNA level for each of the 4 candidate genes in postnatal day 1 (P1) cortex (Table S1), consistent with the time of development of these vessels [31]. *Itgal* did not display allele-specific differential gene expression (Figure S5), consistent with a collateral-*independent* effect on infarct volume. We also noted that neither *Fam57b* nor *Qprt* showed allele-specific expression, and we were unable to detect *4933440M02Rik* in either P1 or adult cortices. These other genes are therefore also unlikely to play a role in infarction via effects on collateral vessel anatomy.

### Increased mRNA level of a splice variant of *Itgal* corresponds with allelic variation between mouse strains

**Figure 6:**
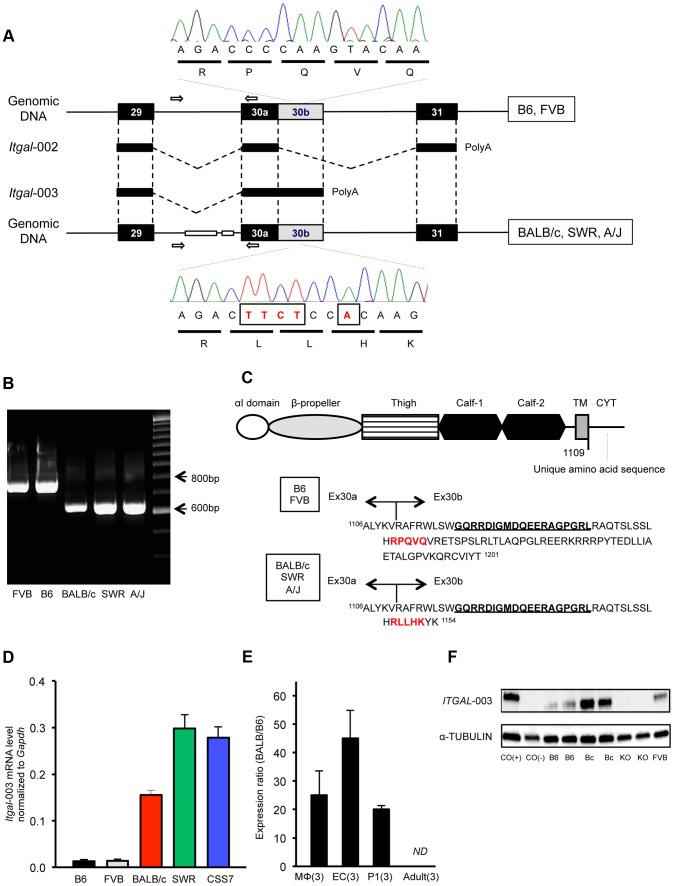
A truncated cytoplasmic tail of the protein and increased mRNA level of a splice variant of *Itgal* correspond with allelic variation between mouse strains. A. Schematic of the *Itgal*-003 transcript showing sites of the ∼150-bp deletion in intron 29 and a 5-bp insertion (TTCT and A) in the coding region of exon 30b, present in BALB/c, SWR, and A/J strains. The 5-bp insertion causes a premature termination of the protein, resulting in a shorter cytoplasmic tail of the protein. B. Allelic variation of the ∼150 bp deletion in intron 29 in 5 mapping strains is confirmed by PCR of genomic DNA, with primer pairs (white arrow in A). C. Amino acid sequences of the cytoplasmic tail of the *Itgal*-003 protein. The 5-bp insertion in exon 30b results in an *Itgal*-003 protein that is identical to the canonical protein, except a short stretch of new amino acid sequence in the truncated cytoplasmic tail. The 5-amino acid sequence in red is matched with the electropherograms in (A). The 19 amino acids (GQRRDIGMDQEERGPGRL) bolded and underlined are used as an epitope to generate an *Itgal*-003-specific polyclonal antibody. D. mRNA levels of *Itgal*-003 transcripts (ENSMUST00000120857) in P1 cortex from B6, FVB, BALB/c, SWR/J, and CSS7 strains as determined by qRT-PCR. Each bar represents mean ± SEM. E. The allele-specific expression ratio of *Itgal*-003 in F1 mice. The level of BALB/c-specific *Itgal*-003 transcript was approximately 26 times, 44 times, and 19 times higher than the B6-specific transcript in macrophages (MΦ), endothelial cells (EC) and P1 cortex, respectively. *Itgla*-003 was not detected in adult cortex (ND). Each bar represents the allele-specific expression ratio (BALB/B6) of the *Itgal* transcript averaged for three F1 animals. F. Western blot for an epitope specific to the *Itgal*-003 encoded protein. Lysates of P1 brain of B6, BALB/c, *Itgal* knockout (KO) and FVB mice. Cos (+), the recombinant *Itgal* protein generated by transfection of COS-7 cells with *Itgal*-003 cDNA and Cos (−), MOCK transfected COS-7 cells with pEYFP plasmid.

While performing the complete re-sequencing of all of the coding exons (including at least 50 bp of flanking intron) for the 4 candidate genes, we found that the large infarct strains, BALB/c, SWR, and A/J, harbor a complex rearrangement in the distal region of the *Itgal* gene; a ∼150 bp deletion in intron 29 and multiple insertions and deletions (indels) in the coding and 3′-UTR of exon 30b of an alternative splice form of *Itgal* (*Itgal*-003, ENSMUST00000120857) (Figure 6A,B). Further sequencing of cDNA of the *Itgal*-003 transcript identified a 5-bp insertion in the coding region of exon 30b in BALB/c, SWR, and A/J strains causing a frameshift in the encoded protein, resulting in novel amino acid sequence and a shorter cytoplasmic tail of the protein, as compared to strains B6 and FVB (Figure 6C).

We also found that the mRNA level of *Itgal*-003 markedly differed between the 5 mapping strains in the P1 cortex. The mRNA level of this splice variant was substantially higher (>11-fold) in the large infarct strains (BALB/c, SWR, and CSS7) than that of the small infarct strains, B6 and FVB mice (Figure 6D). Using the more accurate allele-specific expression analysis, the level of BALB/c *Itgal*-003 transcript is approximately 20-fold higher than B6 transcript in P1 cerebral cortex in F1 (B6×BALB/c) animals (Figure 6E), indicating that sequence variation in *cis* leads to increased levels of the *Itgal*-003 transcript in the BALB/c strain. Since the interplay between neurons, endothelial cells, and glial cells plays a crucial role in the early development of the neurovascular unit, and thus, the pathogenesis of cerebral ischemia [4], we investigated the mRNA profile of *Itgal*-003 in both CD146 (LSEC)-positive endothelial cells and CD11b-positive brain macrophages isolated from F1 embryos (E18.5). As illustrated in Figure 6E, BALB/c-specific *Itgal*-003 transcript is expressed 43 times and 25 times higher in endothelial cells and macrophages, respectively, than the B6-specific transcript in the allele-specific expression analysis. Interestingly, the *Itgal*-003 transcript was not detected in the adult cerebral cortex by RT-PCR, suggesting that this splice variant is acting primarily or exclusively during brain development. To date, 5 *Itgal* splice isoforms have been identified in mice (Figure S6), so we determined the relative allele-specific expression of all the other *Itgal* splice isoforms. No difference was found in P1 and adult cerebral cortices for the other isoforms (data not shown). To further determine whether the strain specific level of *Itgal*-003 transcript is also reflected at the level of the protein, we generated an *Itgal*-003-*specific* antibody against the unique cytoplasmic tail peptide to assay protein expression (Figure 6C). Consistent with the mRNA level, western blot analysis of P1 cerebral cortex demonstrated markedly increased protein level in strain BALB/c compared to strains B6 and FVB (Figure 6F).

### *Itgal* is neuroprotective for ischemic brain damage

**Figure 7:**
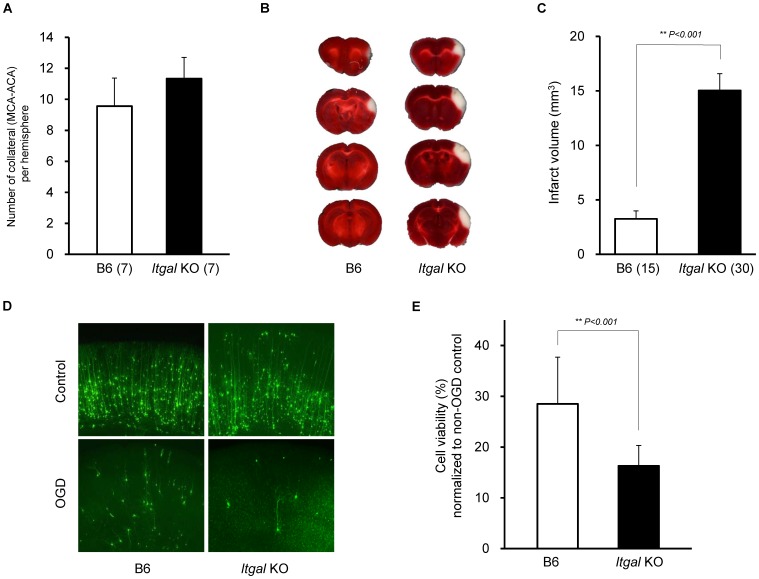
Infarct volume is increased in *Itgal* knockout (KO) mice. A. Collateral number does not differ between B6 and *Itgal* KO mice. B. The posterior faces of representative 1-mm coronal sections from B6 and *Itgal* KO mice are shown. The size of the infarct in *Itgal* KO is larger than B6 mice. C. *Itgal* KO mice on the B6 background exhibit infarct volume ∼3-fold larger than that of B6 mice (15.9 vs. 4.7 mm^3^). ** *P<0.001*. D. Increased neuronal death in the cortical region of the brain slices from *Itgal* KO mice compared to control B6 mice 24 hr after 5.5 min OGD. E. Deficiency of *Itgal* increases neuronal cell death by ∼50% compared to B6 mice. Values represent mean ± SEM from at least 5 animals per group. ** *P<0.001*.

To determine whether *Itgal* is involved in ischemic brain injury *in vivo*, we next examined the phenotype of *Itgal* knockout mice [32]. A recent study reported that there was no difference in collateral vessel number or in infarct volume between *Itgal* knockout and control B6 mice [33]. However, despite also finding no difference in number of collateral arteries (Figure 7A), we observed that infarct volume in *Itgal* knockout mice (n = 30) of B6 background (17 generations backcrossed into B6) was ∼3-fold larger than that observed in B6 mice (15.5 mm^3^ vs. 4.7 mm^3^) (Figure 7B,C). We believe that this difference in infarct volume results from the use of different surgical techniques. When performing permanent distal MCAO, care has to be taken to position the occlusion proximal to the lenticulo-striate branches [34], [35]. If the artery is occluded more distally, smaller and more variable infarcts are produced. In support of this explanation for the difference between the studies, they report a difference in infarct volume of ∼3-fold between B6 and BALB/c strains [33], whereas we routinely observe that infarct volume in BALB/c mice is ∼7-fold larger than that of B6 mice (4.7 mm^3^ vs. 34.2 mm^3^) (Figure 1B).

**Figure 8:**
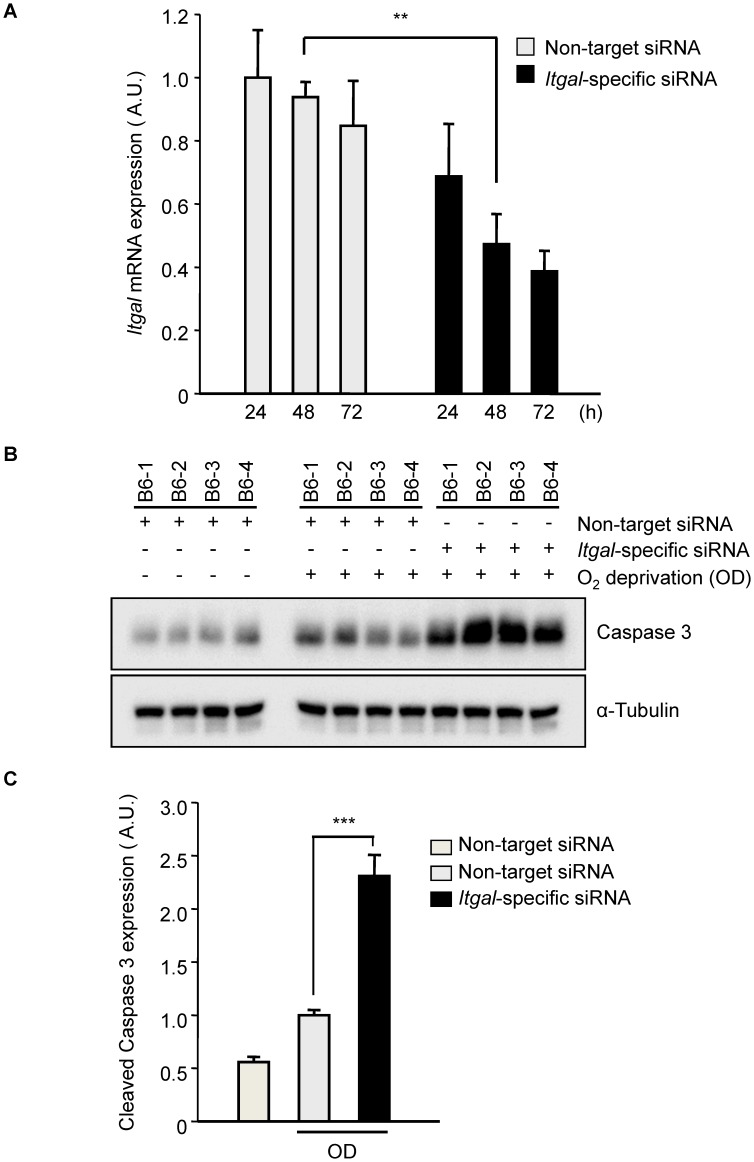
Oxygen deprivation increases cleaved Caspase 3 in an *ex vivo* brain slice stroke model. A. Efficiency of *Itgal* knock-down in *ex vivo* brain slices using siRNA. Non-target or *Itgal*-specific siRNA were transfected into cortical brain slices from B6 mice for 3 days. *Itgal* mRNA levels normalized to *Gapdh* control were determined by qRT-PCR. Values represent the mean±SEM of 4 mice.***P<0.01. B. Western blots were performed for cleaved Caspase-3 in explanted brain slices transfected with either non-target siRNA or *Itgal*-specific siRNA in both control and oxygen deprivation (OD) conditions. C. Quantification of panel B. Cleaved Caspase-3 expression level was normalized to an alpha-tubulin control. Values represent the mean±SEM of 4 mice. *** *P<0.001*.

Since the *Civq1* locus retaining infarct volume phenotype displays both vascular and non-vascular neuroprotective effects on ischemic tissue injury, we hypothesized that the increased infarct volume in *Itgal* knockout mice might be related to neuroprotection after focal cerebral ischemia. To examine this further, we determine the level of OGD-induced neuronal cell death in brain slices from *Itgal* knockout mice, again counting YFP-transfected cortical pyramidal neurons in brain slices. As a control, there was no difference in YFP-transfection efficiency and viability in non OGD-treated brain slices between B6 and *Itgal* knockout mice (Figure S2). Consistent with the infarct volume data, *Itgal* knockout mice showed an approximately 50% increase in neuronal cell death after transient OGD, compared to control B6 mice (Figure 7D,E). After OGD treatment, the level of cleaved Caspase-3 was also significantly increased in lysates of brain slices from *Itgal* knockout mice compared to B6 mice (Figure S3). To further investigate whether reduced levels of *Itgal* modulate ischemic brain injury, and particularly whether these effects were collateral vessel-independent, we employed an *ex vivo* model of cerebral ischemia, using siRNA to knockdown *Itgal* expression in cortical brain slices (where collateral circulation is no longer relevant). Consistent with the increased neuronal cell death observed in *Itgal* knockout mice, after transient oxygen deprivation, *Itgal* siRNA-treated brain slices from B6 mice show markedly increased levels of cleaved Caspase-3, a marker of neuronal cell death (Figure 8). These results indicate that *Itgal* plays a protective role in ischemic brain damage, independent of tissue reperfusion through the collateral vessels.

It should be noted that the *Itgal* knockout mouse line used in our study was generated by replacing the exons 1 and 2 with a Neo-cassette [32] but more recent data shows that there are 5 known alternative splicing transcripts of the gene, including transcripts that do not include these two exons (Figure S6). Thus, we examined whether this *Itgal* knockout mouse line generates null alleles for all of the splice variants of the gene. We found that a splicing variant that uses a different initiation site (*Itgal*-004, ENSMUST00000118405) was detected in P1 and adult cortices by RT-PCR (Figure S6), suggesting that the phenotypes we observed in the knockout mice represent only partial knockout of the entire complement of *Itgal* gene transcripts/functions. Thus, this well-established *Itgal* knockout line may retain residual or additional isoform protein functions, and in the sense of total *Itgal* gene function(s), thus represents a hypomorphic allele.

The *Itgal* knockout allele was generated using 129Sv (Stevens) genomic DNA and a 129S7 (129S7/SvEvBrd-Hprt^b-m2^) ES cell line. Thus, it is formally possible that the some of the differences in infarct volume that are seen in the *Itgal* knockout were contributed by 129 alleles flanking the deleted *Itgal* locus. Although the original strains used in the knockout construction are not widely available, we have determined infarct volume and collateral artery number for the related 129S1/SvImJ strain which is derived from the original 129/Sv strain [36]. Both infarct volume and collateral artery number of 129S1/SvImJ mice are not significantly different from those of B6 mice (Figure S7). Thus, we conclude that the effects seen with the *Itgal* KO mice are *primarily* due to the loss of the *Itgal* gene, and not linked (129Sv or 129S7) polymorphisms, although we cannot rule out modest bystander effects.

## Discussion

Although several approaches have been proposed to reduce ischemic brain damage, including reperfusion, neuroprotection, and neuronal regeneration, for the vast majority of stroke patients, current therapies are limited. We reasoned that given the multiplicity of mechanisms causing cell death in stroke, approaches that augment endogenous protective pathways might be more likely to lead to success. Thus, we have attempted to identify novel genetic factors modulating stroke outcomes by exploiting naturally occurring endogenous genetic variation determining ischemic brain injury. In crosses between inbred mouse strains that exhibit large differences in infarct volumes, we previously identified a QTL (*Civq1*) mapping to distal chromosome 7 that determines more than 50% of the variation observed between the strains. In this study, we present evidence that *Itgal* is one of the genes underlying the complex *Civq1* locus.

Despite the almost routine detection of QTLs for important disease traits in both rodents and humans, identification of causal genes underlying QTLs remains a major obstacle, in large part due to the large confidence intervals for the typical QTL, often covering hundreds of candidate genes [26]. To narrow the *Civq1* interval we employed three different methods that capitalize on the structure of the mouse genome: (1) generation of interval-specific congenic lines, (2) genome wide association (EMMA) analysis across more than 30 inbred mouse strains, and (3) interval-specific SNP haplotype analysis using the 5 inbred strains from our experimental crosses. The latter approach was most effective at reducing the list of candidate genes in the *Civq1* interval to only 4 genes that clearly fall within shared SNP haplotype blocks. Of these 4 genes, *Itgal* was the only gene that harbored non-synonymous coding SNPs and exhibited altered mRNA abundance, with both molecular phenotypes co-segregating with the infarct volume phenotypes in the 5 strains used in QTL mapping. We also found that allelic variation in an alternative splicing variant of *Itgal* (*Itgal*-003) resulted not only in differential transcript abundance, but also in a truncated cytoplasmic tail of the protein, consisting mostly of a novel amino acid sequence.

*Itgal* encodes the α subunit of LFA-1(α_L_β_2_) integrin, which is highly expressed in microglia, spleen, bone marrow, and most immune cell populations. The binding of intracellular proteins to the cytoplasmic tail of *Itgal* is essential to the activation of LFA-1 integrin. The canonical splice isoform of *Itgal* (*Itgal*-002, ENSMUST00000117762) contains this important functional domain, conserved among all integrin family members. Mutation of the cytoplasmic tail of *Itgal* (*Ital-002*) has been shown to inhibit its interactions with intracellular proteins, destabilize integrin conformation, and disconnect to cytoskeleton [37], [38]. By contrast, the function of the unique, truncated sequence of the cytoplasmic tail found in *Itgal*-003 remains unknown. The increased expression of the *Itgal*-003 in large infarct strains may interfere with the functions of the well-studied, reference isoform of *Itgal*, or with other α subunits (α_D_, α_M_, and α_X_) that can bind to the β_2_ subunit, resulting in inhibition of cell adhesion and migration during the development and/or tissue injury. In addition to these differences in the cytoplasmic tail of *Itgal-003*, the inbred mouse strains show two amino acid substitutions (W972R and P978L) in the calf-2 domain that segregate with the infarct volume phenotypic difference. These two coding changes in ITGAL fall in relatively poorly conserved residues of the protein and the 972R BALB/c allele is shared with other species including cow, sheep, and opossum. However, the lack of conservation at these residues itself does not allow us conclude that these changes are inconsequential because non-synonymous coding SNPs causing risk for complex genetic traits tend not to fall within highly conserved residues/regions of proteins [39]. In point of fact, W972R is predicted to be deleterious by multiple *in silico* amino acid substitution databases. Although the exact role of the calf-2 domain is not fully understood, a point mutation in the calf-2 domain of α_v_β_3_ integrin in Glazmann thrombasthenia patients disrupts the normal contacts between α and β subunits, resulting in impaired cellular transport from the endoplasmic reticulum [40]. Thus, the two amino acid substitutions in the calf-2 domain of ITGAL may have an effect on α-β formation and stabilization of LFA-1 integrin. Taken together, natural DNA sequence variation in the mapping strains in *Itgal* generates both qualitative (a strain-specific splice variant and two amino acid coding changes) and quantitative (overall transcript level) changes in the transcript and encoded protein. We do not know exactly which of these changes is most relevant to the infarct volume phenotype but we surmise that it is likely to be a combination of some or all of these. Importantly, we have shown that a congenic animal that retains all of these strain-specific differences in *Itgal* variation from B6 shows a similar phenotype to B6 mice, whereas the *Itgal* gene knockout appears similar to BALB/c strain. In addition, siRNA knockdown of *Itgal* in an *ex vivo* cerebral ischemia model using cortical brain slices from B6 mice also increases the extent of neuronal cell death. These combined data suggest that in comparison to the B6 *Itgal* allele, the net effect of the BALB/c allele is at least a *partial* loss of function.

The identification of *Civq1* has raised the question of the role of the causative gene(s) in pathophysiology of ischemic stroke. A previous study suggested that the differential ischemic outcomes between inbred mouse strains is related to intrinsic differences in ischemic tolerance or protection pathways in neural tissue [7]. More recently, the extent of pial collateral circulation in the brain has been shown to be inversely correlated with infarct volume data between 15 inbred strains [41]. The authors also identified a QTL (*Canq1*) for collateral vessel number mapping to the identical genomic position as *Civq1*, thereby proposing that the causative gene controlling collateral circulation might also determine the differential infarct volume [21]. Surprisingly, we have observed that the genetically isolated *Civq1* locus retaining infarct volume phenotype displays both vascular (collateral circulation) and non-vascular (neuroprotection) effects in the modulation of ischemic brain damage after MCAO. These observations are in accord with our previous study that this locus displays a non-vascular protective effect on ischemic insult in skeletal muscle [42]. In a mouse model of hind-limb ischemia, we previously mapped a strong genetic locus determining limb necrosis and recovery of perfusion (*Lsq1*) at the identical genomic position as *Civq1* on chromosome 7 [43]. Using an *in vitro* model of hypoxia and nutrient deprivation, where collateral circulation and indeed all circulation is absent, we have found that isolated primary myocytes from BALB/c are more sensitive to hypoxia and nutrient deprivation than B6 myocytes, recapitulating the strain-specific response to hind-limb ischemia. More importantly, muscle cells from the congenic mice (C.B6-*Civq1*-3) are protected from this same *in vitro* hypoxic insult, indicating that the B6 allele(s) of the causative gene(s) plays an important role in survival of muscle cells, independent of any vascular contribution to ischemia [42]. Therefore, given the physiological similarities between the two ischemic models and the identical map position, we propose that the same causative gene(s) underlying *Civq1*/*Lsq1* determines ischemic tissue damage in multiple tissues, possibly through the same physiological mechanism.

A number of studies have demonstrated that microglia play an important protective role in ischemic brain damage through microglial migration to the site of injury [44], [45], which is controlled by the *Itgal* protein [46], [47]. Microglial cells with down-regulated *Itgal* expression fail to protect neurons after OGD in cultured hippocampal brain slices, suggesting that the migration and adhesion of microglial cells regulated by *Itgal* is important for the beneficial effect of microglia in stroke [47]. These published data further support *Itgal* as one of the genes underlying *Civq1*.

However, *Itgal* also shows deleterious effects in stroke, as *Itgal* is also involved in inflammatory injury after cerebral ischemia. After the ischemic insult, the brain is invaded by blood-circulating leukocytes, and LFA-1 (containing *Itgal*) regulates the interaction between circulating leukocytes and endothelial cells. Deficiency of *Itgal* shows a protective effect on ischemia-reperfusion injury using a *transient* MCAO model [48]. However, it should be stressed that *permanent* (our study) and *transient* MCAO [48] models exhibit different pathophysiologies [49]. Large numbers of circulating blood cells enter the brain at time points later than 24 hr after the ischemic insults [50], [51], but we measure infarct volume at 24 hr after MCAO. Thus, microglia, expressing *Itgal*, may play their critical, *protective* role in the early stages of stroke.

Thus far, we have emphasized vascular-*independent* functions of *Itgal* in the modulation of infarct volume. But it is clear that the *Civq1* locus also contains genetic determinants that modulate infarct volume via changes in collateral vessel anatomy. The identity and nature of these genes and gene variants remains to be determined. Recent studies have demonstrated that microglia regulate vascular anastomosis and increase vascular complexity by assisting endothelial tip cell fusion during brain development [52]. LFA-1 (containing *Itgal*) modulates adhesion of monocyte to collateral endothelium involved in arteriogenesis [53], [54]. Given the important role of microglia and *Itgal* in these processes related to collateral vessel development, the question remains then why the *Itgal* knockout mouse line used in this study did not exhibit a collateral vessel phenotype. Because an engineered knockout allele is rarely equivalent to a naturally-occurring variant allele at a QTL [14] and an *Itgal* splice isoform was in fact detected in these *Itgal* “knockout” mice, we cannot exclude the possibility that collateral vessel development and neuroprotection are genetically regulated by different alleles or splice isoforms of *Itgal*.

Alternatively, it is quite likely that more than one gene underlies the complex *Civq1* locus. One or more genes may modulate infarct volume by their effect on neuroprotection or inflammatory physiology, and another gene or genes may modulate infarct volume by regulating collateral artery formation. Recent successes in QTL gene identification support this conjecture. Multiple, physically linked, smaller effect genes often contribute to the overall effect of robust, large effect QTLs [14], [55]–[58]. In this light, we note that a number of gene products mapping within the *Civq1* interval have well-defined roles in the immune response (cytokine receptors, integrins), tissue remodeling (MMP21, ADAM12), or metabolism (cytochrome c oxidase), each of which could be involved in the overall response to ischemia. Generation of sub-congenic strains that further divide the *Civq1* locus may help identify these genes.

Although the *Itgal* haplotype across 32 inbred strains generally correlates with infarct volume, the correlation falls off for several outlier strains. For example, despite that fact that the NOD strain shares the BALB/c haplotype for the *Itgal* locus, this strain exhibits a small infarct volume and its mRNA expression level is significantly higher than those of the large infarct strains (Figure S8). Similarly, strain C3H shares the B6 haplotype including a high mRNA expression level, the W972 and P978 SNPs, and lacks the genomic deletion, but the C3H infarct volume is larger than most of the small infarct strains. These data suggests for certain outlier strains, loci other than the otherwise large-effect *Civq1* locus can cause profound phenotypic effects. In line with this hypothesis, we have identified a novel genetic locus mapping to mouse chromosome 8 in an intercross between B6 and one of these outlier strains, C3H [59]. Overall, these results are consistent with what we have shown from our previous mapping data, namely, that genetic variation in *Itgal* is not solely causative for the differential phenotype. Even within the *Civq1* locus, there are additional genes for determining infarct volume (ie, a gene(s) regulating collateral vessel density). Nonetheless, our data using congenic and *Itgal* knockout mice show that at least one of the causative gene(s) underlying *Civq1* functions in the survival of brain tissue independent of a vascular contribution to the ischemic response. Taken together, these data show that the extent of the collateral circulation will not be the sole mechanism underlying *Civq1*/*Lsq1*, and possibly for other loci as well.

In summary, by showing evidence of its role in regulation of ischemic brain injury in a mouse model of stroke, we have identified *Itgal* as one of possibly many quantitative trait genes for *Civq1*. Natural DNA sequence variation in *Itgal* generates qualitative and quantitative change of the transcript and the encoded protein, and a knockout allele, even while retaining one of the newly described splice isoforms, shows a robust effect on infarct volume *in vivo* and *in vitro*. Future studies using cell type-specific knockout mice will help dissect cellular and molecular mechanisms of *Itgal* in both vascular and non-vascular contributions to ischemic brain injury. Ultimately, this work will provide insight into the endogenous protective pathways involved in the pathophysiology of ischemic tissue damage, and in the long-term, provide novel targets for potential therapeutic intervention of ischemic stroke.

## Materials and Methods

### Ethics statement

All experiments were performed under protocols approved by the Animal Care and Use Committee of Duke University.

### Animals

All inbred strains and *Itgal* knockout mice (B6.129S7-*Itgal* ^tm1Bll^/J) were obtained from the Jackson Laboratory (Bar Harbor, Me) either directly or bred locally from breeding pairs of each strain. Mice were age-matched (12±1 week) for all experiments.

### Permanent distal MCAO stroke model

Focal cerebral ischemia was induced by direct occlusion of the distal MCA as described previously [8]. Briefly, mice were anesthetized with ketamine (100 mg/kg) and xylazine (5 mg/kg), and the right MCA was exposed by a 0.5-cm vertical skin incision midway between the right eye and ear. After the temporalis muscle was split, a 2-mm burr hole was drilled at the junction of the zygomatic arch and the squamous bone. While visualizing with a stereomicroscope, the right MCA was cauterized using an electrocauterizer (Fine Science Tools). The cauterized MCA segment was then transected with micro scissors to verify that the occlusion was complete. The surgical site was closed with 6-0 sterile nylon sutures, and 0.25% bupivicaine was applied. Animals were maintained at 37°C during and after surgery until fully recovered from anesthesia, when they were returned to their cages and allowed free access to food and water. All mice were housed in an air-ventilated room with ambient temperature maintained at 25±0.5°C.

### Measurement of infarct volume

Twenty-four hours after surgery, the animal was euthanized and the brain was removed, chilled at −80°C for 3 min to slightly harden the surface and sliced into 1-mm coronal sections using a brain matrix. Slices were stained with 2% TTC (2,3,5-Triphenyl-tetrazolium chloride) as previously described [8]. Infarct volumes were calculated by measuring infarct areas on the separate slices, multiplying areas by slice thickness, and summing all slices; this “indirect” morphometric method corrects for edematous swelling.

### Collateral artery measurement

Under ketamine (100 mg/kg) and xylazine (5 mg/kg), the left ventricle of the heart was cannulated. The right atrium of the heart was incised to allow for venous outflow and the circulation was cleared and maximally dilated with heparin (50 µg/ml), adenosine (1 mg/mL) and papaverine (40 µg/mL) in phosphate-buffered saline (PBS). Immediately after the PBS infusion, the skull and dura were carefully removed and blue polyurethane (PU4ii, Vasqtec) with a viscosity sufficient to restrict capillary transit (1∶1 resin∶methylethyl ketone) was injected. Formalin (10% in PBS) was applied topically to the cortex, and the polyurethane was allowed to cure for 20 minutes. After post-fixation in 10% formalin overnight, the pial circulation was imaged (Leica MZ16FA). Analysis was confined the measurement of collaterals between the MCA and ACA trees [41].

### OGD of brain slices

Brain slice isolation and OGD experiments were performed as previously described [22], [23]. B6, BALB/c, C.B6-*Civq1*-3, and *Itgal* KO mice were euthanized at postnatal day 10. Each brain was cut into 250 µm coronal slices on a Vibratome (Vibratome) in chilled culture medium containing 15% heat-inactivated horse serum, 10 mM KCl, 10 mM HEPES, 100 U/ml penicillin/streptomycin, 1 mM MEM sodium pyruvate, and 1 mM L-glutamine in Neurobasal A supplemented with NMDA inhibitor (1 µM MK-801). Brains were divided into “hemi-coronal” slices. For OGD, slices were suspended at 34°C for 5.5 min in glucose-free, N_2_-bubbled artificial CSF containing 140 mM NaCl, 5 mM KCl, 1 mM CaCl_2_, 1 mM MgCl_2_, 24 mM D-glucose, and 10 mM HEPES. Control and OGD-treated brain slices were plated onto a 12-well plate with solid culture medium made by the addition of 0.5% agarose. After explanting the brain slices, plates were placed for recovery at 37°C for 30 min in a humidified incubator under 5% CO_2_. Gold particle-coated plasmids containing Yellow Fluorescent Protein (YFP) were introduced into the brain slices by biolistic transfection using a Helios Gene Gun (Bio-Rad, Herculues). Slice cultures were maintained at 37°C for 24 hr in humidified incubator under 5% CO_2_.

### Genotyping

Single nucleotide polymorphism (SNP) genotyping was performed using the GoldenGate genome-wide mouse 377 SNP panel (Illumina). Genomic positions of genetic markers (NBCI Build37/mm9) were retrieved from the UCSC genome browser (http://www.genome.ucsc.edu/) and converted to cM using the mouse map converter (http://cgd.jax.org/mousemapconverter). Additional SNP (rs13479513, rs13468481, rs45999701, rs32420445, rs37240399 and rs6216320) and microsatellite (D7Mit281, D7Mit238, and D7Mit68) markers were used for fine-mapping of the locus. The distal boundary of the critical interval (9.6 Mb) of C.B6-*Civq1*-3 was determined by locations of maximal breakpoints for the genotyped markers. The critical interval was homozygous for BALB/c alleles at 126.2 Mb (D7Mit238) and at 135.82 Mb (rs45999701).

### Linkage analysis

Genome-wide scans were plotted using the J/QTL mapping program, version 1.2.1 (http://research.jax.org/faculty/churchill/). Suggestive (*P = 0.63*) and significant (*P = 0.05*) thresholds were established empirically for each phenotypic trait by 1,000 permutation tests using all informative markers. The percentage of total trait variance attributable to each locus was determined using the Fit-QTL function provided within the J/QTL software.

### Genome-wide association EMMA analysis

Genome-wide association mapping for infarct volumes for the 32 strains was performed with EMMA using the UCLA web-based server (http://mouse.cs.ucla.edu/emmaserver) [12]. Analysis of individual phenotypic data was performed with SNP panels consisting of 4 million SNPs [11]. We determined confidence intervals by expanding the interval around the peak SNP to include all neighboring SNPs surpassing the significance threshold (*P* = 10^−5^). For single SNP associations, the QTL confidence interval was set at 3 Mb (1.5 Mb kb on either side of the peak SNP). SNP-associated P values were transformed with −log10 (P value) for graphing association scores.

### Interval-specific SNP haplotype analysis

For the 9.6 Mb interval on chromosome 7, SNP data were obtained from the Mouse Phenome Database (http://phenome.jax.org/), the Wellcome Trust Sanger Institute Mouse Genome Browser (http://www.sanger.ac.uk/cgi-bin/modelorgs/mousegenomes/snps.pl), and the Center for Genome Dynamics (http://cgd.jax.org). Physical map position was based on the genomic sequence from the NCBI Build 37/mm9. Haplotype blocks were defined as three or more adjacent informative SNPs shared between the large infarct strains (BALB/c, A/J and SWR/J) which differed from the haplotype for the small infarct strains (B6 and FVB) [25].

### Generation and purification of the anti-*Itgal*-003 antibody

The cytoplasmic tail peptide (GQRRDIGMDQEERAGPGRL) of *Itgal*-003 was synthesized and then used to generate an *Itgal*-003-specific polyclonal antibody (Bethyl Laboratories). The rabbit antiserum was affinity purified and tested for immunoreactivity for *Itgal-003* by immunoblotting.

### Isolation of embryonic macrophages and endothelial cells

Each E18.5 embryonic brain was cut into small pieces, incubated in DMEM containing 10% fetal bovine serum and Collagenase type IV (0.2 mg/ml, Sigma) for 30 min at 37°C and then passed through a 19G syringe to obtain a homogeneous cell suspension. Cells were washed with PBS supplemented with 0.5 mM EDTA and 0.5% BSA and incubated in 10 µl anti-CD131 (Endothelial cells) or anti-CD11b (Macrophages) antibody-conjugated magnetic beads (Miltenyi Biotech) for 15 min on ice. These cells were applied to MACS MS separation columns on magnetic stands and washed with 1.5 ml PBS supplemented with 0.5 mM EDTA and 0.5% BSA. The column was removed from the magnetic stand and magnetically labeled cells were isolated by flushing out fractions. Isolated cells were used to extract total RNA using Trizol (Invitrogen).

### SYBR_quantitative RT-PCR

Quantification of RT-PCR products were measured by examining the increase in fluorescence that was induced by SYBR green binding to dsDNA (Applied Biosystems). The reaction was analyzed on an ABI 7700 Sequence Detection system using the following conditions: 95°C for 10 minutes followed by 40 cycles of 95°C for 15 seconds and 60°C for 1 minute. All samples were run in triplicate and additional assays for endogenous controls (*Gapdh* and/or *Hprt1*) were performed to control for input cDNA template quantity. Relative quantification was determined for each sample by calculating the mean Ct value using the 2^−**ΔΔ**Ct^ method. Sequence detection software (SDS version 2.1.1, Applied Biosystems) was used for this analysis.

### Allele-specific SNapShot gene expression analysis

This approach requires at least one SNP in the transcript to distinguish the alleles of the two strains. PCR was performed using an appropriate dilution of cDNA generated from the cerebral cortex of F1 (B6×BALB) animals. Amplicons containing coding SNPs were amplified by conventional PCR, and 15 ul of PCR products were treated with 1 U exonuclease I (New England Biolabs) and 5 U of Shrimp Alkaline Phosphatase (SAP) (Promega). Purified PCR products were used in combination with a conventional primer designed to sit at the nucleotide to the immediate 5′ position of a coding SNPs in the transcript. Cycling conditions were as follows: 40 cycles of 95C° for 10 s, 50C° for 5 s, and 60C° for 30 s. The primer is then labeled with a fluorescently tagged dideoxynucleotide through a single base pair extension (SNaPshot kit from ABI). These products were treated with 1 U of SAP prior to running on an ABI 3130 sequencer, and peak heights were determined using Gene Mapper software (ABI). To determine conditions under which the SNaPshot assays were quantitative, genomic DNA from the F1 animals was amplified and also analyzed by SNaPshot using the same extension. The expression ratio of each transcript allele was normalized to the ratio of the two alleles in the F1 genomic DNA.

### *Itgal* siRNA knock-down experiments

To reduce *Itgal* mRNA expression by RNA interference, the *Itgal*-specific siRNA was purchased from Thermo Scientific and used as recommended. For the *ex vivo* stroke experiments, siRNA was delivered to the cortical brain slices before the oxygen deprivation. Briefly, after explanting brain slices from B6 mice (age P10), plates were placed for recovery at 37°C for 30 min in a humidified incubator under 5% CO_2_. 5 µM of non-target pooled (D-001910-10-05) or *Itgal*-specific pooled (E-046772-00-0005) siRNAs were introduced onto the brain slices and the slices were maintained at 37°C in a humidified incubator under 5% CO_2_. Forty eight hours after introducing the siRNA, brain slices were deprived of oxygen using glucose-free, N_2_-bubbled artificial CSF. The slices were then incubated for an additional 24 hr.

### Flow cytometry

Mice were perfused with PBS and brains were dissected out and weighed. Brains were teased apart and digested for 1 hour at 37C with 2 mg/mL collagenase A (Roche) and 0.25 mg/mL DNase I (Roche). Cells were strained and centrifuged in a 30% over 70% Percoll (Invitrogen) in PBS density gradient. Cells from the interface were isolated and red blood cells were lysed with ACK lysis buffer. Cells were counted and stained with the following antibodies: CD11a-PE or IgG2a-PE (eBioscience); CD11c-PE-Cy5.5 (eBioscience); CD45-PE-Cy7 (eBioscience); Ly6G-AF700 (eBioscience); IA-IE-Qdot655 (eBioscience); and CD11b-BV780 (Biolegend). Flow cytometry was run on a LSR-II in the Duke Human Vaccine Institute Flow Research Facility. Analysis was done with FlowJo (Treestar).

### Western blot analysis

Following anesthesia, the whole brains were isolated from mice, and were homogenized in cold lysis buffer (50 mM Tris/HCl [pH 7.4], 1 mM EGTA, 1 mM DTT) containing a protease and phosphatase inhibitor cocktail (Thermo Scientific). After low speed centrifugation (1,000×g, 5 min), supernatants (30 ug) were then electrophoresed in a 4–12% polyacrylamide gel and electro-blotted for 2 hr on PVDF (Polyvinylidine Fluoride) membranes at room temperature. The blot was incubated with the polyclonal anti-*Itgal*-*003* (1∶1,000) or anti-alpha-Tubulin (1∶5,000) primary antibodies overnight at 4C°. To detect the level of apoptosis, six brain slices from each group were collected after OGD treatment. Anti cleaved-Caspase3 antibody (1∶3,000, Cell Signaling Tech) was applied to detect level of apoptotic cell death. The protein bands were visualized using the chemiluminescence reaction (ECL detection kit).

### Statistical analysis

Results were represented as the mean±SEM. Statistical analysis of infarct volume, number of primary branch from MCA as well as collateral vessel number were performed using 1-way ANOVA or non-parametric Kruskal–Wallis test. For qRT-PCR and SNapShot allele-specific expression analyses, student's t-test was used to determine if the fold change was significantly different.

## Supporting Information

Figure S1 Genotype-phenotype correlation for infarct volume in an F2 intercross between FVB and BALB/c strains. The plots display the phenotypic effect of the allele at SNP rs13479513 on infarct volume in the F2 cohort.
(TIF)

Figure S2 Cell viability curve in B6, BALB/c, Line-3(C.B6-*Civq1*-3), and *Itgal* KO mice. Total numbers of healthy and YFP-positive neurons in the cortical region of the brain slices were counted for 6 days after slice preparation under non-OGD conditions.
(TIF)

Figure S3 Level of apoptotic cell death in cortical brain slices from B6, BALB/c, Line3 (C.B6-*Civq1*-3), and *Itgal* KO mice. Western blots were performed to detect cleaved Caspase-3 in explanted brain slices from non-OGD control and OGD conditions. Caspase-3 expression level was normalized to alpha-tubulin control. Values represent mean±SEM from at least 5 animals per group (* P<0.05, ***P<0.001).
(TIF)

Figure S4 Non-synonymous coding SNPs in the *Qprt* and *Itgal* genes. Alignments of portions of each protein sequence are compared across different inbred mouse strains and other mammalian species. The positions of the relevant amino acid residues are indicated and sources of sequences are shown on the left. Gray boxes indicate no differences between B6 or FVB and the other species. (A) The amino acid residues at position 205 and 253 of *Qprt* are not conserved, although Glutamine (Q) at position 205 is conserved in all mammalian species except rodents. (B) Structural schematic of lymphocyte function-associated antigen-1 (LFA-1, α_L_β_2_). The two coding SNPs that create W972R and P978L polymorphisms located in calf-2 extracellular domain in α_L_ integrin. (C) The amino acid residue at position 972 of ITGAL variable among mammalian species, but tryptophan is found only in the small infarct mouse strains (B6 and FVB) and in the horse. The Proline found at amino acid position 978 in B6 and FVB mouse strains is conserved in some mammalian species, but variable in others.
(TIF)

Figure S5 *Fam57b* and *Qprt* do not exhibit strain-specific differences in message RNA levels. (A) mRNA levels of *Fam57* were determined by qRT-PCR in P1 cortex from the 5 mapping strains. No significant expression difference between the strains was detected. (B) The allele-specific *Qprt* transcript level ratio in three F1 (B6×BALB) mice. The non-synonymous SNP (rs33122161, G/A) in exon 2 was used to detect the G-allele (B6) and A-allele (BABL/c) transcripts of the *Qprt* gene in embryonic macrophages (Φ), endothelial cells (EC), P1 and adult cerebral cortices of F1 mice. Each bar represents the ratio of the two parental transcript alleles normalized to the signal obtained for the genomic DNA of F1 animals. The expression level of the BALB-specific transcript was only slightly lower (∼0.86×) than that of the B6-specific transcript in the tissues.
(TIF)

Figure S6 (A) Differential splicing of the *Itgal* gene produces 5 possible transcript isoforms. The commonly used *Itgal* KO mouse line was generated by insertion of a Neo-cassette into the genomic region harboring exons 1 and 2. (B) Identification of the cDNA variant *Itgal-004* by RT-PCR (primers: white arrows) in P1 and adult cortices in the *Itgal* KO and B6 mice.
(TIF)

Figure S7 Both collateral artery number of 129S1/SvImJ mice (A) and infarct volume (B) of 129S1/SvImJ and 129X1/SvJ mice are not different from those of B6 mice.
(TIF)

Figure S8 (A) W972R and P978L non-synonymous coding SNPs, (B) the ∼150-bp deletion in intron 29 and (C) mRNA level of *Itgal* do not perfectly segregate with the volume of cerebral infarction across inbred strains.
(TIF)

Table S1 Primer sequences.
(TIF)
