# Supplementary figures and images for: Natural Genetic Variation of Integrin Alpha L (Itgal) Modulates Ischemic Brain Injury in Stroke
Source: PLoS Genet. 2013 Oct 10;9(10):e1003807. doi: 10.1371/journal.pgen.1003807 (PMC3794904; doi:10.1371/journal.pgen.1003807)

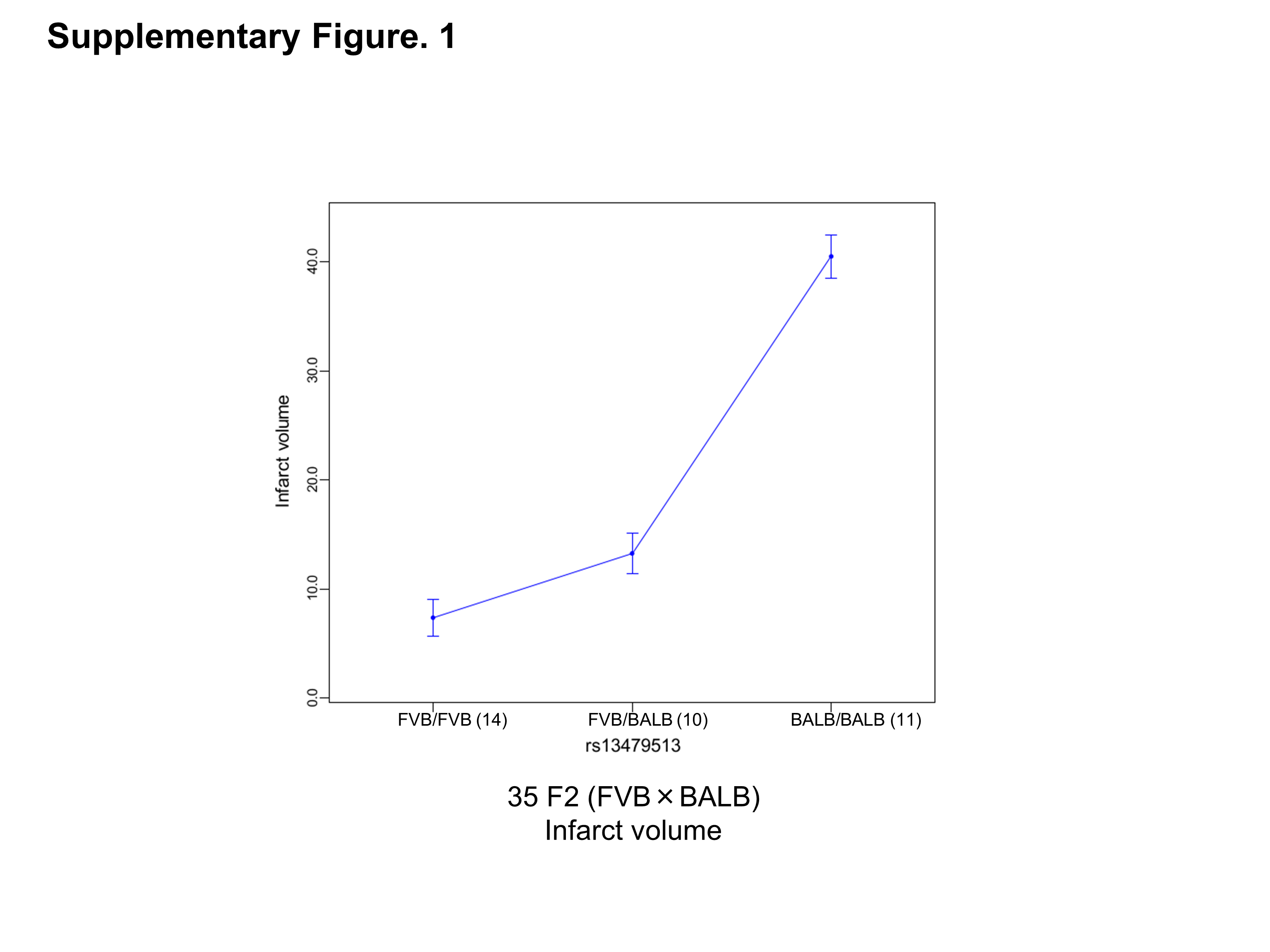

Supplement: Figure S1 — Genotype-phenotype correlation for infarct volume in an F2 intercross between FVB and BALB/c strains. The plots display the phenotypic effect of the allele at SNP rs13479513 on infarct volume in the F2 cohort. (TIF) [file pgen.1003807.s001.tif]

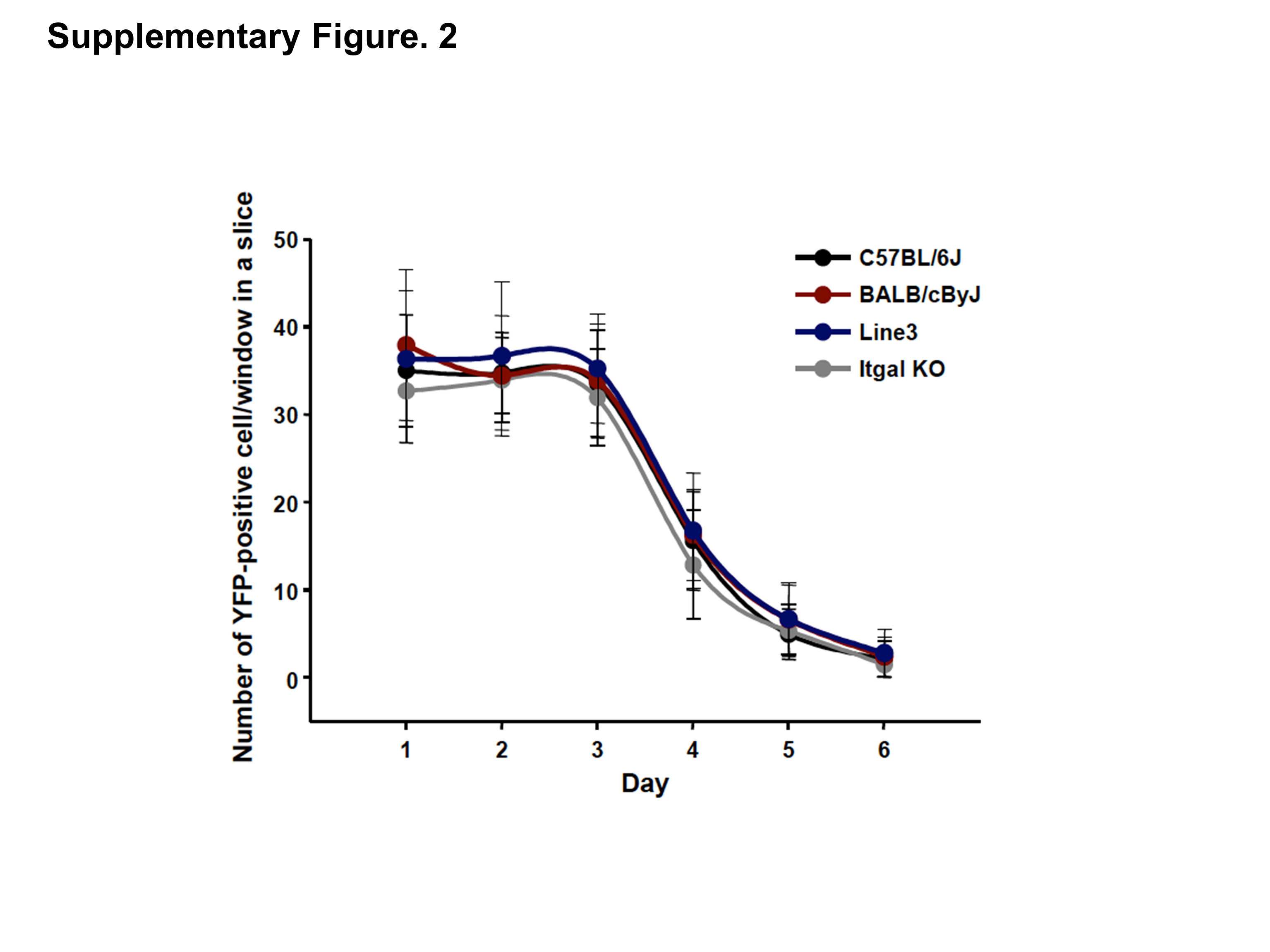

Supplement: Figure S2 — Cell viability curve in B6, BALB/c, Line-3(C.B6-Civq1-3), and Itgal KO mice. Total numbers of healthy and YFP-positive neurons in the cortical region of the brain slices were counted for 6 days after slice preparation under non-OGD conditions. (TIF) [file pgen.1003807.s002.tif]

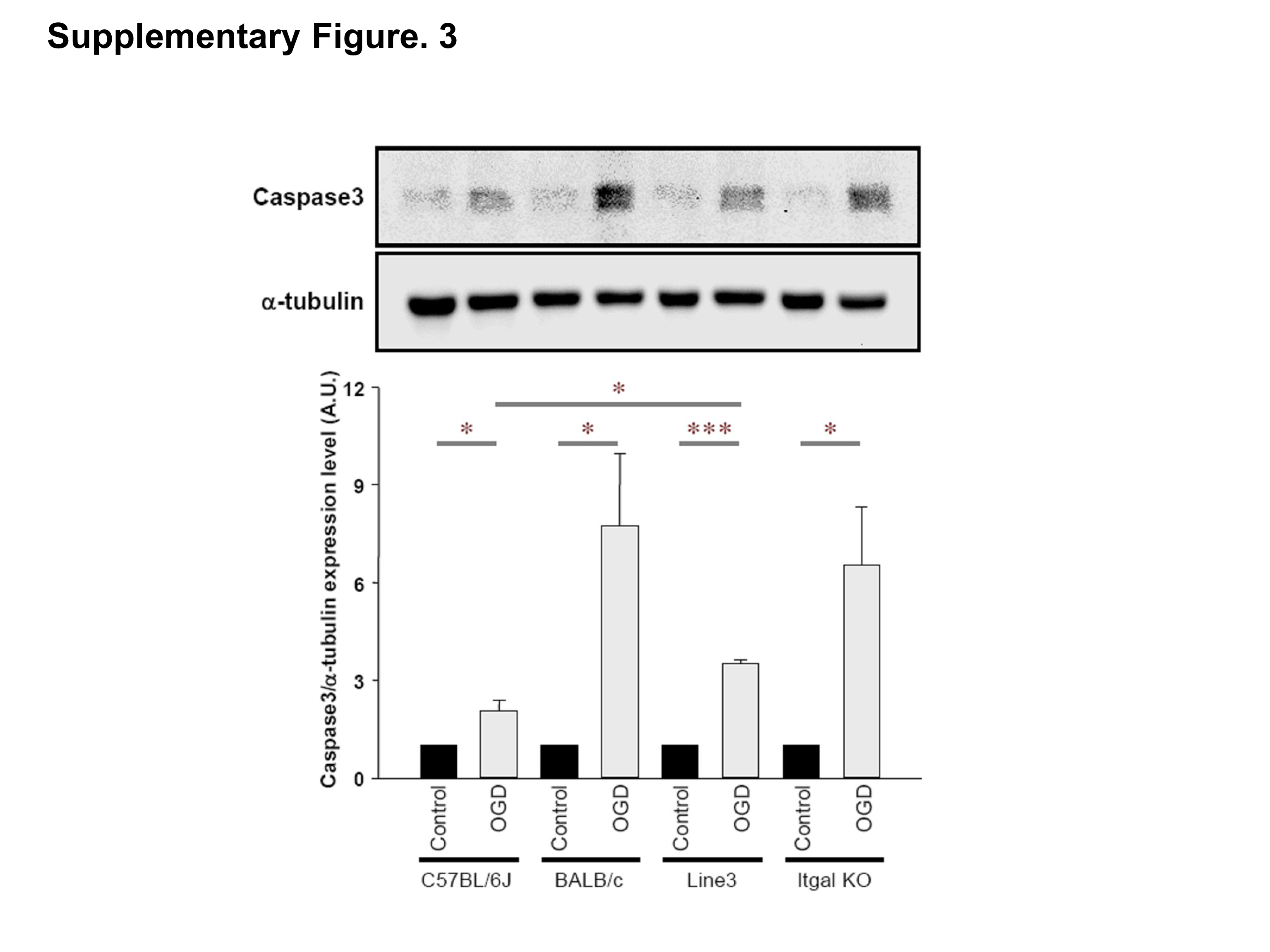

Supplement: Figure S3 — Level of apoptotic cell death in cortical brain slices from B6, BALB/c, Line3 (C.B6-Civq1-3), and Itgal KO mice. Western blots were performed to detect cleaved Caspase-3 in explanted brain slices from non-OGD control and OGD conditions. Caspase-3 expression level was normalized to alpha-tubulin control. Values represent mean±SEM from at least 5 animals per group (* P<0.05, ***P<0.001). (TIF) [file pgen.1003807.s003.tif]

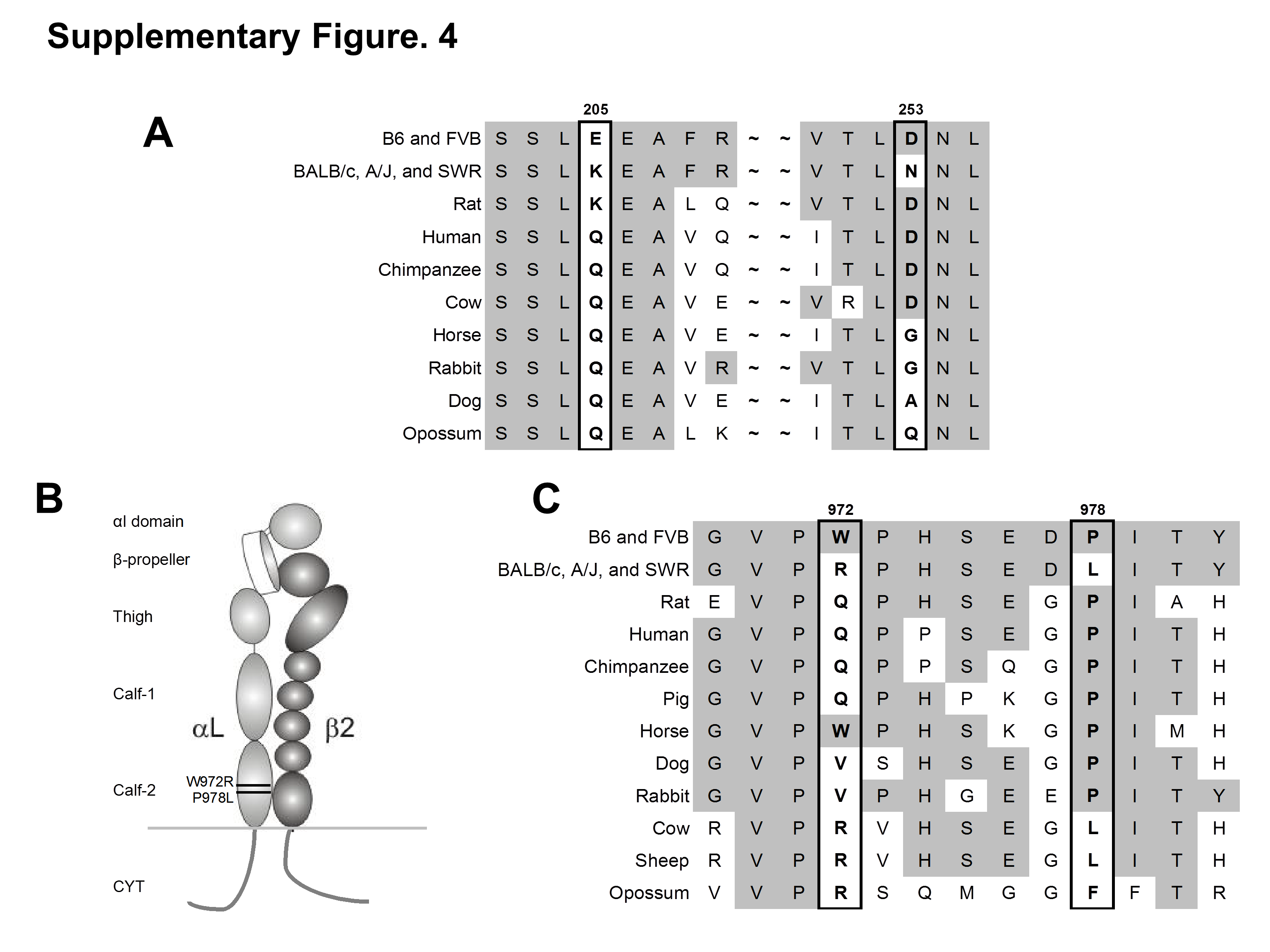

Supplement: Figure S4 — Non-synonymous coding SNPs in the Qprt and Itgal genes. Alignments of portions of each protein sequence are compared across different inbred mouse strains and other mammalian species. The positions of the relevant amino acid residues are indicated and sources of sequences are shown on the left. Gray boxes indicate no differences between B6 or FVB and the other species. (A) The amino acid residues at position 205 and 253 of Qprt are not conserved, although Glutamine (Q) at position 205 is conserved in all mammalian species except rodents. (B) Structural schematic of lymphocyte function-associated antigen-1 (LFA-1, αLβ2). The two coding SNPs that create W972R and P978L polymorphisms located in calf-2 extracellular domain in αL integrin. (C) The amino acid residue at position 972 of ITGAL variable among mammalian species, but tryptophan is found only in the small infarct mouse strains (B6 and FVB) and in the horse. The Proline found at amino acid position 978 in B6 and FVB mouse strains is conserved in some mammalian species, but variable in others. (TIF) [file pgen.1003807.s004.tif]

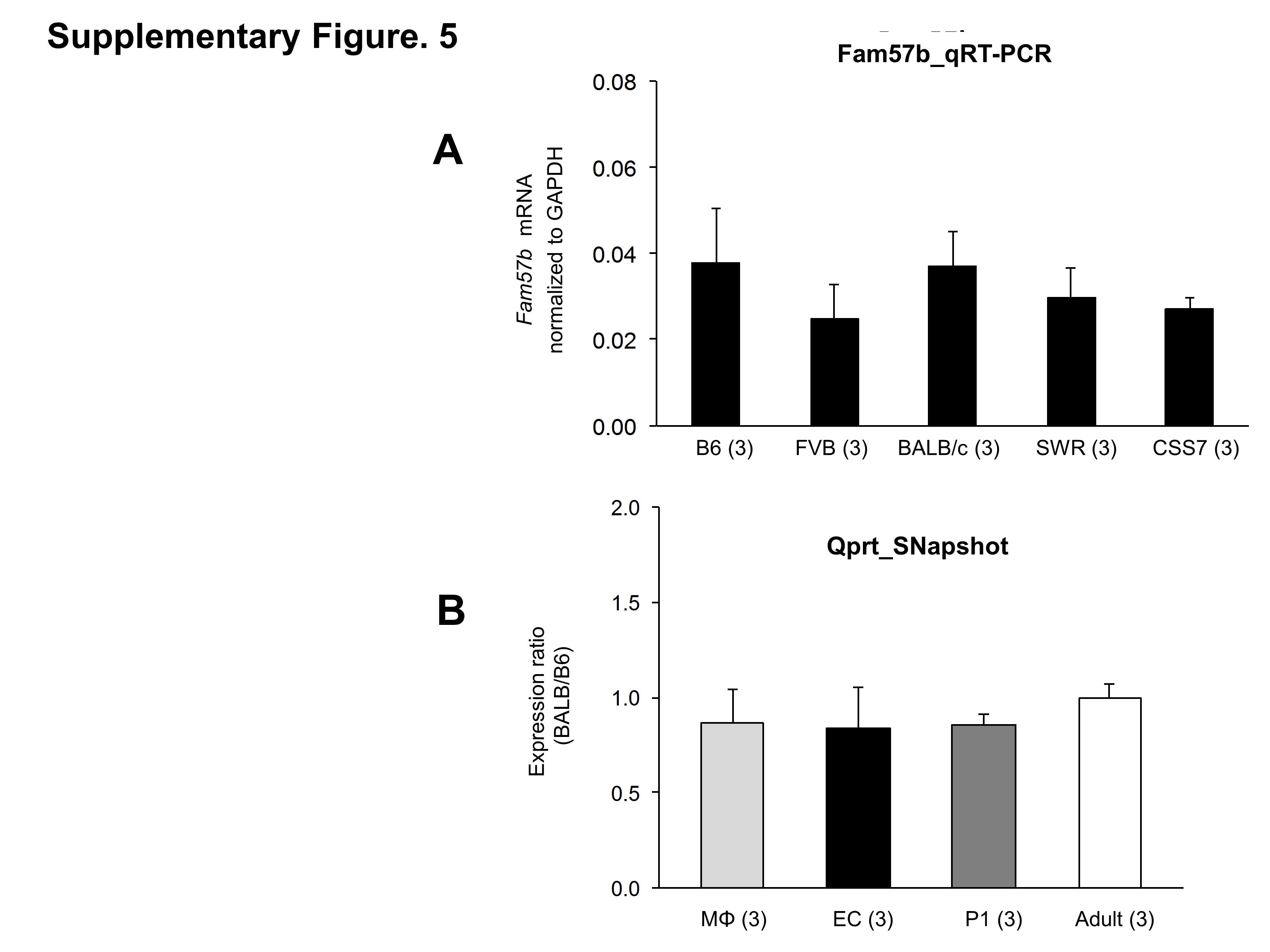

Supplement: Figure S5 — Fam57b and Qprt do not exhibit strain-specific differences in message RNA levels. (A) mRNA levels of Fam57 were determined by qRT-PCR in P1 cortex from the 5 mapping strains. No significant expression difference between the strains was detected. (B) The allele-specific Qprt transcript level ratio in three F1 (B6×BALB) mice. The non-synonymous SNP (rs33122161, G/A) in exon 2 was used to detect the G-allele (B6) and A-allele (BABL/c) transcripts of the Qprt gene in embryonic macrophages (Φ), endothelial cells (EC), P1 and adult cerebral cortices of F1 mice. Each bar represents the ratio of the two parental transcript alleles normalized to the signal obtained for the genomic DNA of F1 animals. The expression level of the BALB-specific transcript was only slightly lower (∼0.86×) than that of the B6-specific transcript in the tissues. (TIF) [file pgen.1003807.s005.tif]

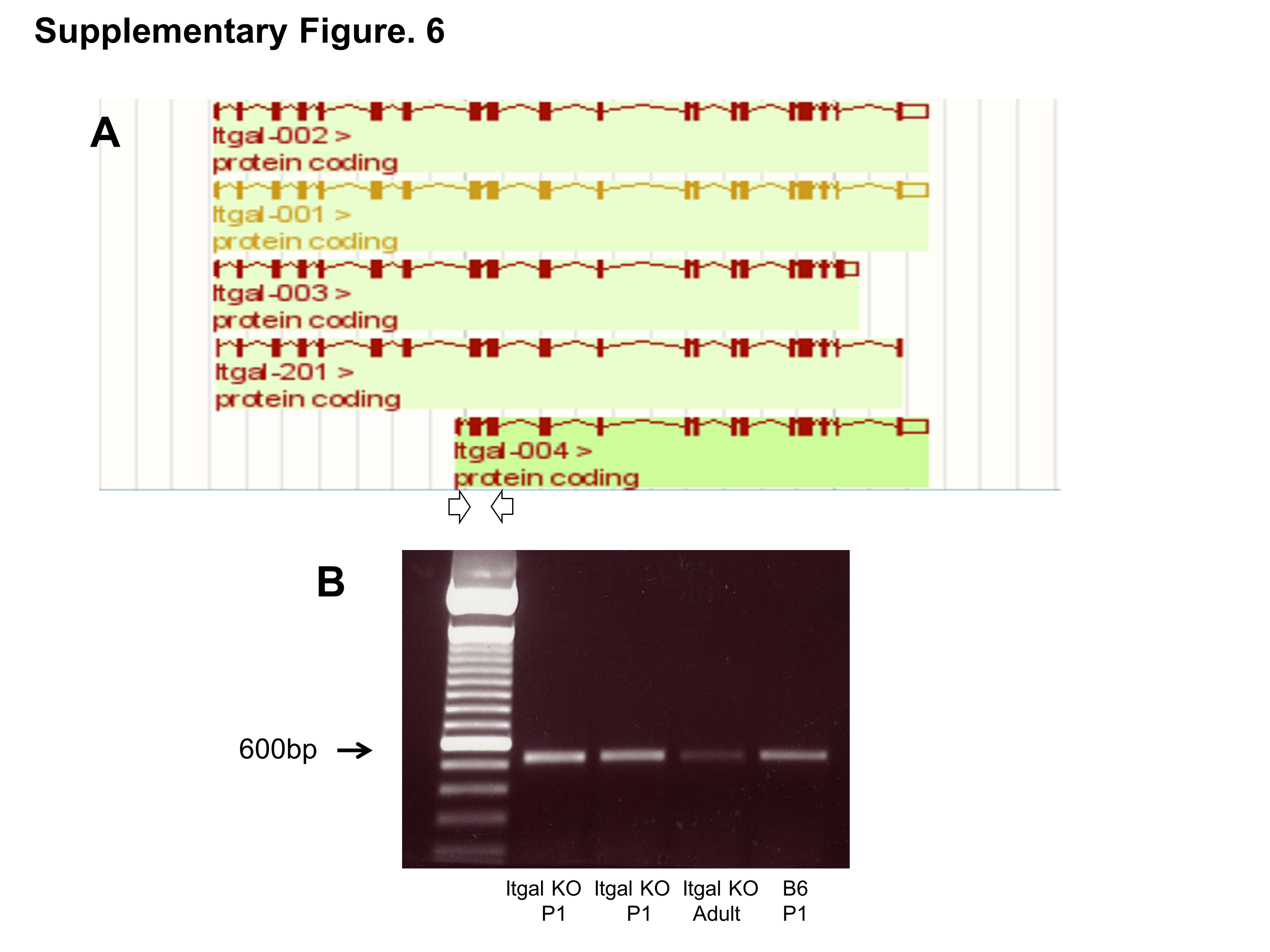

Supplement: Figure S6 — (A) Differential splicing of the Itgal gene produces 5 possible transcript isoforms. The commonly used Itgal KO mouse line was generated by insertion of a Neo-cassette into the genomic region harboring exons 1 and 2. (B) Identification of the cDNA variant Itgal-004 by RT-PCR (primers: white arrows) in P1 and adult cortices in the Itgal KO and B6 mice. (TIF) [file pgen.1003807.s006.tif]

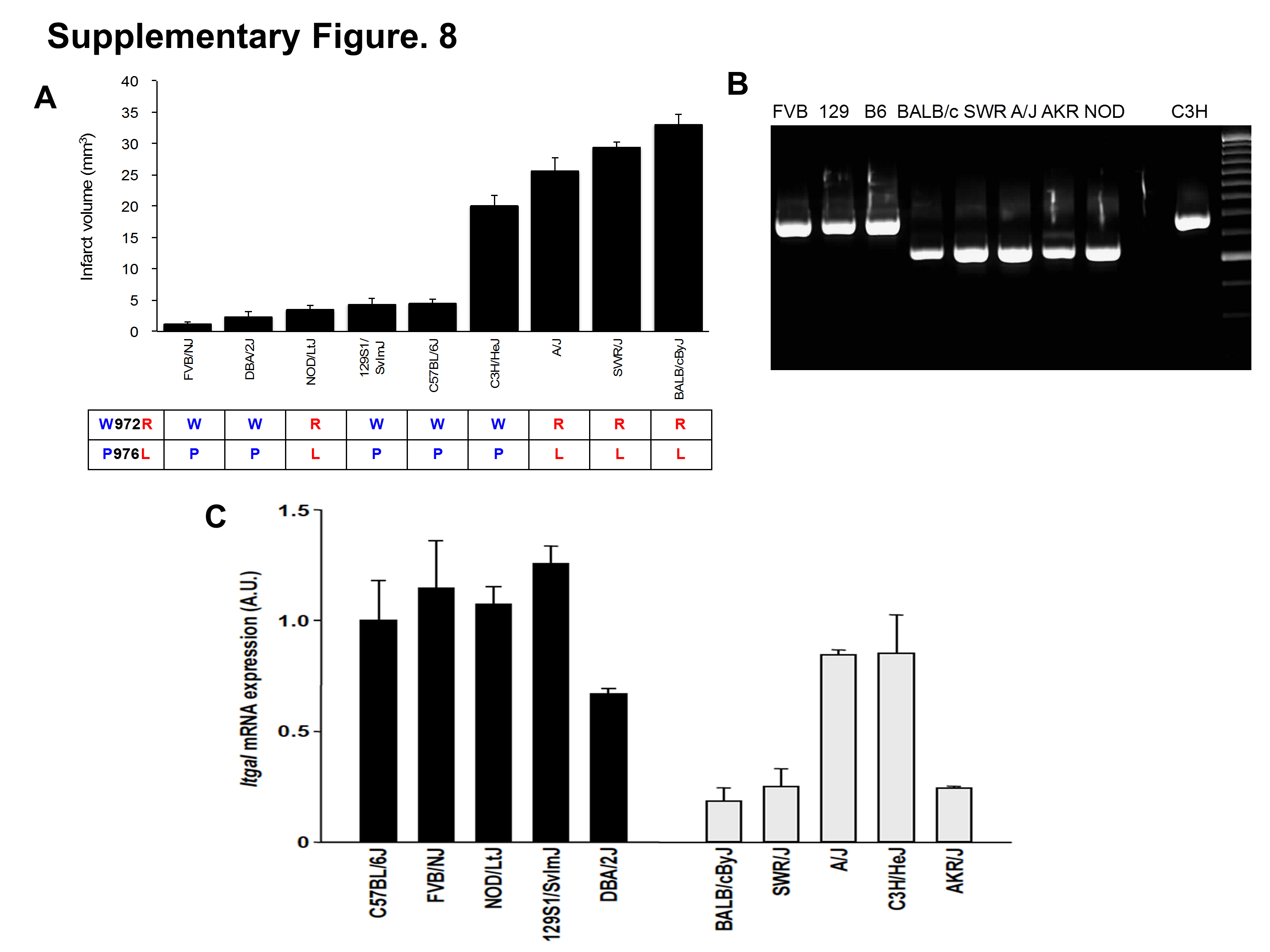

Supplement: Figure S8 — (A) W972R and P978L non-synonymous coding SNPs, (B) the ∼150-bp deletion in intron 29 and (C) mRNA level of Itgal do not perfectly segregate with the volume of cerebral infarction across inbred strains. (TIF) [file pgen.1003807.s008.tif]

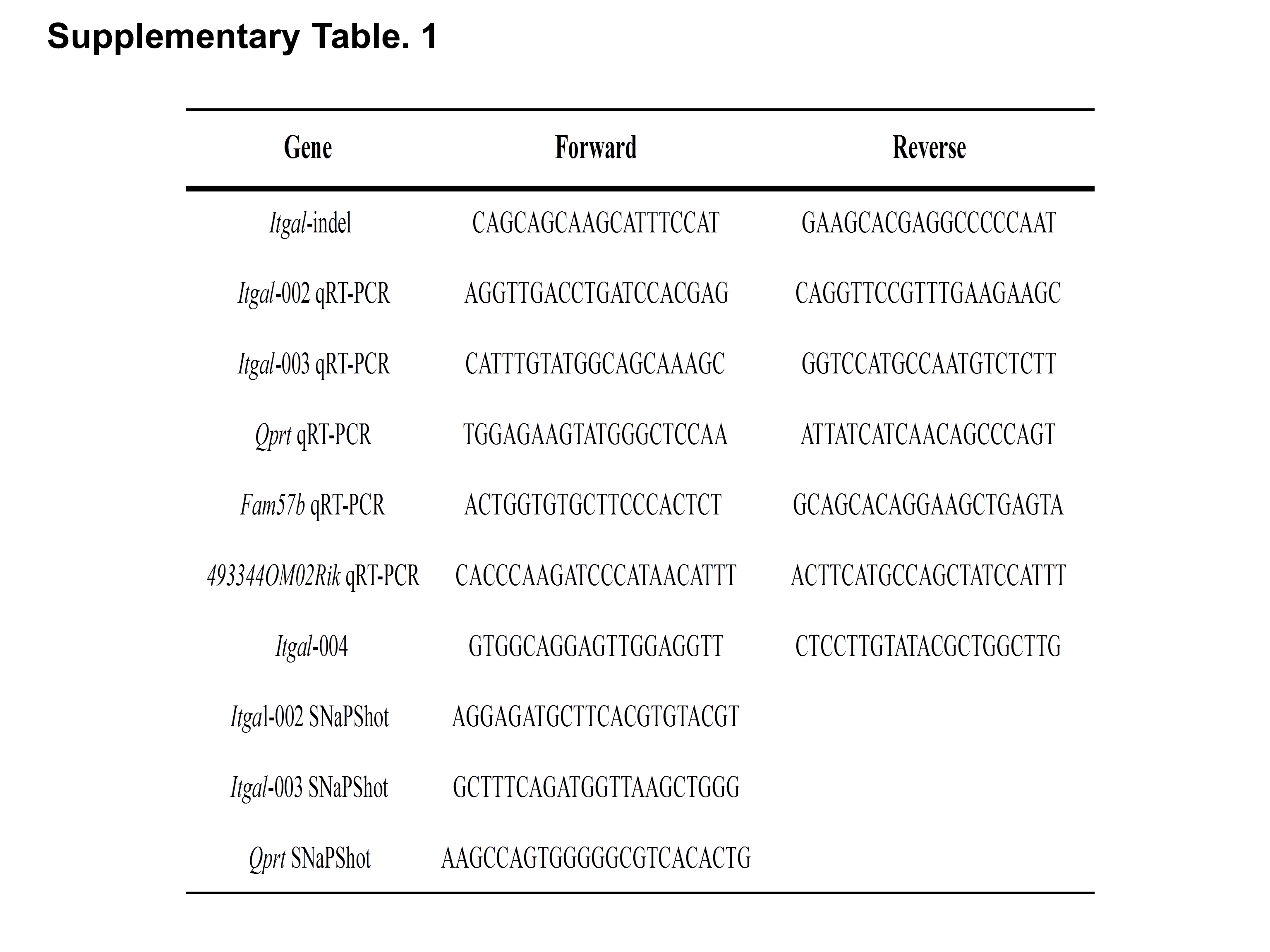

Supplement: Table S1 — Primer sequences. (TIF) [file pgen.1003807.s009.tif]
